# The T_H_1 cell lineage-determining transcription factor T-bet suppresses T_H_2 gene expression by redistributing GATA3 away from T_H_2 genes

**DOI:** 10.1093/nar/gkac258

**Published:** 2022-04-19

**Authors:** Arnulf Hertweck, Maria Vila de Mucha, Paul R Barber, Robert Dagil, Hayley Porter, Andres Ramos, Graham M Lord, Richard G Jenner

**Affiliations:** UCL Cancer Institute and Cancer Research UK UCL Centre, University College London (UCL), London, WC1E 6BT, UK; UCL Cancer Institute and Cancer Research UK UCL Centre, University College London (UCL), London, WC1E 6BT, UK; UCL Cancer Institute and Cancer Research UK UCL Centre, University College London (UCL), London, WC1E 6BT, UK; Comprehensive Cancer Centre, School of Cancer & Pharmaceutical Sciences, King's College London, London, SE1 1UL, UK; Research Department of Structural and Molecular Biology, University College London, Darwin Building, Gower Street, London, WC1E 6XA, UK; UCL Cancer Institute and Cancer Research UK UCL Centre, University College London (UCL), London, WC1E 6BT, UK; Research Department of Structural and Molecular Biology, University College London, Darwin Building, Gower Street, London, WC1E 6XA, UK; Faculty of Biology, Medicine and Health, University of Manchester, Manchester, M13 9NT, UK; UCL Cancer Institute and Cancer Research UK UCL Centre, University College London (UCL), London, WC1E 6BT, UK

## Abstract

Lineage-determining transcription factors (LD-TFs) drive the differentiation of progenitor cells into a specific lineage. In CD4^+^ T cells, T-bet dictates differentiation of the T_H_1 lineage, whereas GATA3 drives differentiation of the alternative T_H_2 lineage. However, LD-TFs, including T-bet and GATA3, are frequently co-expressed but how this affects LD-TF function is not known. By expressing T-bet and GATA3 separately or together in mouse T cells, we show that T-bet sequesters GATA3 at its target sites, thereby removing GATA3 from T_H_2 genes. This redistribution of GATA3 is independent of GATA3 DNA binding activity and is instead mediated by the T-bet DNA binding domain, which interacts with the GATA3 DNA binding domain and changes GATA3′s sequence binding preference. This mechanism allows T-bet to drive the T_H_1 gene expression program in the presence of GATA3. We propose that redistribution of one LD-TF by another may be a common mechanism that could explain how specific cell fate choices can be made even in the presence of other transcription factors driving alternative differentiation pathways.

## INTRODUCTION

Cell fate choice is determined by lineage-determining transcription factors (LD-TF). LD-TFs often antagonise each other's expression and function in order to drive mutually exclusive gene expression programmes. However, differentiating cells frequently express more than one LD-TF but the effect that this has on the function of the factors and on cell fate choice is often unclear (1–5).

Mutually antagonistic LD-TFs are frequently co-expressed in CD4^+^ helper T cells (1,4–6). Naïve CD4^+^ helper T cells respond to antigen and cytokine cues by differentiating into one of a number of specialised effector cell lineages (7–10). This functional segregation allows the development of tailored immune responses against specific types of pathogens. The classical example of mutually exclusive effector programs is the differentiation of CD4^+^ T cells into the T_H_1 or T_H_2 lineage in response to intracellular viral and bacterial infections and extracellular parasites, respectively (11,12). Precise control of effector cell lineage determination is critical for an effective immune response and inappropriate T cell differentiation may result in inefficient pathogen clearance or immune pathology (6,13–15).

T_H_1 and T_H_2 cell differentiation are driven by the LD-TFs T-bet (TBX21) and GATA3, respectively. Robust differentiation of each lineage is attained through transcription factor and cytokine-mediated positive feedback loops and by cross-inhibition of the alternative cell fate. Specifically, T-bet directly activates the gene encoding the T_H_1 subtype-defining cytokine interferon gamma (IFNγ) and represses genes encoding the T_H_2 cytokines IL-4, IL-5 and IL-13, while GATA3 displays the opposite functionality (16–27). Furthermore, both IFNγ and IL-4 function in an autocrine manner to further stabilise the corresponding lineage. In addition, both LD-TFs repress the other's expression (28) and can bind and auto-activate their own genes (29,30).

These positive and negative feedback loops theoretically generate a bistable switch between T_H_1 and T_H_2 cell states. However, T-bet and GATA3 are co-expressed in *in vitro* differentiated primary human Th1 cells (29,31,32) and in human CCR5^+^ T_H_1 memory cells (33). Furthermore, although *in vitro* polarised murine T_H_2 cells stably maintain a T_H_2 phenotype, transfer into a mouse model of lymphocytic choriomeningitis virus infection leads to co-expression of GATA3 and T-bet and development of T_H_1 properties (T_H_2 + 1 cells) (34). CD4^+^ T cells co-expressing T-bet and GATA3 can also be generated *in vitro* by activating naïve cells in the presence of IFNγ, IL-12 and IL-4 and such T_H_2/1 hybrid cells have been observed *in vivo*, during infections with intestinal helminths such as *Heligmosomoides polygyrus*, *Schistosoma mansoni* and *Strongyloides ratti* in mice and *Strongyloides stercoralis* in humans (35,36). T_H_2/1 hybrid cells generated from naïve T cells were refractory to cell lineage reprograming during exposure to T_H_1 and T_H_2 polarizing cytokines *in vitro* and remained detectable in a helminth infection model for months after pathogen clearance (35). In addition, experimentally boosting the numbers of T_H_2/1 hybrid cells in *H. polygyrus* infected mice increased IFNγ production and parasite fecundity (37). Together, these previous findings demonstrate that T-bet/Sco-expressing cells do not represent uncommitted intermediates, are stable long-term, and can actively modify the nature of an immune response.

In addition to T-bet and GATA3, other pairs of LD-TFs have been found to be co-expressed in CD4^+^ T cells. T-bet can be co-expressed with Bcl6 or Rorγt (38,39), while the regulatory T cell (T_REG_) LD-TF FoxP3 can be co-expressed with Bcl6, T-bet, GATA3 or Rorγt (4,40–56). However, the effect of LD-TF co-expression on the function of the proteins and the impact on the resultant cell phenotype are not well understood. In human T_H_1 cells, GATA3 exhibits a distinct binding profile characterised by loss from T_H_2 genes and gain at T_H_1 genes (29) suggesting that T-bet may alter GATA3′s DNA binding profile when the two factors are co-expressed. To address this hypothesis, we have expressed T-bet and GATA3 alone or together in T cells and measured the effect on the function of the two factors. We show that T-bet interacts with GATA3 and redistributes it from T_H_2 genes to T-bet binding sites at T_H_1 genes, switching T cells from a T_H_2 to a T_H_1 expression program. We propose that direct sequestration of one LD-TF by another may be a common mechanism through which lineage-determination can proceed in cells co-expressing antagonistic LD-TFs.

## MATERIALS AND METHODS

### Cell culture

EL4-GFP, EL4-T-bet, EL4-GFP + Plum, EL4-T-bet + Plum, EL4-GFP + GATA3 and EL4-T-bet + Plum cells were described previously (29,57). EL4 cells expressing mPlum or HA-GATA3 were transduced with FLAG-Tbx21 Y525F-T2A-BSD-IRES-EGFP or FLAG-Tbx21 R163/R164A-T2A-BSD-IRES-EGFP retrovirus and selected with blasticidin as described (29). EL4-GFP + Plum cells were transduced with retrovirus encoding a doxycycline (dox)-inducible FLAG-T-bet construct and selected with G418. Cells were treated with between 0 and 10 μg/ml dox for 48 hours prior to harvesting. HEK293T cells and EL4 cells were cultured in Dulbecco's modified Eagle's medium supplemented with 10% fetal bovine serum, 4 mM L-alanyl-L-glutamine, 25 mM D-glucose, 1 mM sodium pyruvate, 10 mM HEPES, 100 U/ml penicillin and 100 μg/ml streptomycin. EL4 cells were stimulated with phorbol 12-myristate 13-acetate (50 ng/ml) and ionomycin (1 μM) for 4 hours prior to formaldehyde crosslinking.

### ChIP

Cells were crosslinked by the addition of one-tenth volume of fresh 11% formaldehyde solution for 20 minutes at room temperature before the reaction was quenched by addition of glycine, as described (57). Cells were rinsed twice with 1x PBS and flash frozen in liquid nitrogen. Cells were lysed with non-ionic detergent, the nuclei washed and then lysed with ionic detergent. Cells were sonicated on ice to solubilize and shear crosslinked DNA (27W for 10 × 30 second pulses using a Misonix Sonicator 3000). The resulting whole cell lysate was cleared by centrifugation and then incubated overnight at 4°C with 50 μl of Protein G magnetic Dynabeads that had been pre-incubated with 5 μg of purified antibody (anti-FLAG, M2 Sigma; anti-HA, 3F10 Roche; anti-H3K27ac, ab4729 Abcam). Beads were washed 6 times with RIPA buffer and once with TE containing 50 mM NaCl. Bound complexes were eluted from the beads by heating at 65°C with rocking for 2 hrs and crosslinks then reversed in IP and input DNA by incubation at 65°C for 6 hrs. IP and input DNA were then purified by treatment with RNase A, proteinase K and isolated with KAPA Pure beads.

For ChIP in transiently transfected HEK293T cells, 4.5 × 10^6^ cells were plated onto a 15 cm plate. The cells were transfected 24 hours later with 70 μg of wild-type (WT) HA-GATA3 or HA-GATA3 C320G expression plasmids using polyethyleneimine. The cells were washed with PBS 36 hrs after transfection and crosslinked as above. The remaining steps were carried out as outlined above for EL4 cells except that the cells were sonicated using a Bioruptor Pico (Diagenode) (3 × 30 sec pulses with 30 sec gaps in between).

Specific DNA sequences were quantified in triplicate in ChIP-enriched and input DNA by quantitative PCR (ThermoFisher QuantStudio) using QuantiTect SYBR Green PCR master mix (Qiagen) and enrichment calculated relative to input. Standard curves were performed for all primer pairs to ensure linear amplification. The following primers were used to quantify ChIP enrichment:

*Il4* CNS2 forward: 5′-ATC ACG TCG TCT TAC CCA AAC A-3′,

*Il4* CNS2 reverse: 5′-TGT GGG AGA GCG TCT GAT CTG T-3′,

*Ifng* TSS forward: 5′-CCT GTG CTG TGC TCT GTG G-3′,

*Ifng* TSS reverse: 5′-ACT CCT TGG GCT CTC TGA CG-3′

*Ifng* CNS-6 forward: 5′-GAC GAG CTC TGC AAC CCT TGA AGC TGT GGG TAC-3′,

*Ifng* CNS-6 reverse: 5′-TGA CTC GAG AGA TTG CCG TCT GGT CTT GGC GT-3′

### ChIP-seq

Libraries were constructed from ChIP DNA by standard Illumina protocols, except that DNA in the range 150–350 bp was gel-purified after PCR-amplification. The libraries were quantified using a Qubit fluorometer and Agilent bioanalyzer, pooled and subjected to 50 bp single-end sequencing with a HiSeq 2500 sequencer (except for samples from the dox-induced T-bet experiment, which were subjected to 75 bp single-end sequencing with a NextSeq 550 sequencer). Raw reads were filtered for base quality using FastQC with default parameters and adapters sequences were removed with Trim Galore! Reads passing quality filtering criteria were aligned to GRCm38 using BWA with the default settings (58). Significantly enriched regions were identified with MACS v2 using a significance threshold of q < 0.01 (59). The significance of changes in T-bet and GATA3 binding was assessed with the diffbind R package based on two ChIP replicates (60). The different patterns of GATA3 binding were defined by the following criteria: gain sites: T-bet + GATA3 > GATA3 + GFP at FDR < = 0.05 and called by MACS2 in both T-bet + GATA3 replicates; loss sites: T-bet + GATA3 < GATA3 + GFP at FDR < = 0.05 and called by MACS2 in both GATA3 + GFP replicates; invariant sites: T-bet + GATA3 vs GATA3 + GFP binding ratio < = |1.25|, differentially bound between sample groups at FDR > 0.05 and called by MACS2 in both replicates of both sample groups; all GATA3 sites: called by MACS2 in both replicates of GATA3 + GFP sample. Binding sites that overlapped ENCODE blacklist version 2 regions (https://github.com/Boyle-Lab/Blacklist/tree/master/lists) were removed. Genes whose transcriptional start sites was closest to the center of GATA3 gain, invariant and loss sites were determined with the ChIPpeakAnno R package (61) using the GRCh38 reference transcript assembly. We downloaded previously published T-bet ChIP-seq data from mouse T_H_1 cells (activated with αCD3/CD28 for 3 days followed by 4 days of culture with IL-2 and IL-12: Rep1: GSM998272 (62) and Rep2: GSM836124 (63)) and GATA3 ChIP-seq data from mouse T_H_2 cells (activated with αCD3/CD28 and polarised with IL-4 in the presence of αIFN-γ and αIL-12 for 8 days: GSM2931810 (64)). The data were processed with the same pipeline used for the EL4 data. Only binding sites called by MACS in both T_H_1 cell T-bet ChIP-seq datasets were considered.

Average binding profiles (in reads/million) and heatmaps across sets of T-bet and GATA3 binding sites were generated with ngsplot (65). To control for differences in ChIP efficiency when comparing average profiles between ChIPs of the same factor in different cells, average reads/million across a set of sites were then normalized by the average signal across all sites.

Locations in the human genome (hg19) orthologous to GATA3 binding sites in EL4 cells were identified using liftOver and average GATA3 binding profiles in human T_H_1 cells (GSM776558 (29)) and T_H_2 cells (GSM776559 (29)) plotted across these sites using ngsplot.

### RNA-seq

EL4 cells were stimulated with phorbol 12-myristate 13-acetate (50 ng/ml) and ionomycin (1 μM) for 4 hours and total RNA was then purified with TRIsure (Bioline). Poly-adenylated RNA was isolated with Oligotex (Qiagen) and libraries were prepared using the NEBNext Multiplex Small RNA Library Prep kit and then sequenced on an Illumina HiSeq 2500 using 100 bp paired-end reads. Raw reads were filtered for base quality using FastQC with default parameters and adapters sequences were removed with Trim Galore! Transcript-level read abundances were created with kallisto (66) using the Gencode M20 transcript models and combined with gene-level abundances using tximport (67). Expression estimates were then modelled using the DESeq2 package (68). Ranked gene lists were generated by ordering the expression ratios by -log10 p-value divided by the rank of the fold-change and enrichment for selected gene sets was computed using the fgsea R package (69). T_H_1 and T_H_2 specific gene sets were previously defined (70).

### ATAC-seq

2 × 10^6^ EL4-GFP or EL4-T-bet cells were stimulated with 50 ng/ml PMA and 1 μg/ml ionomycin at 37°C for 4 hrs or left unstimulated. For each ATAC-seq reaction, 50,000 cells were washed in 50 μl cold PBS (500g, 5 min, 4°C) and re-suspended in 50 μl cold lysis buffer I (10 mM Tris-Cl pH 7.4, 10 mM NaCl, 3 mM MgCl2, 0.5% IGEPAL CA-630) and spun (500g, 10 min, 4°C). The supernatant was removed, and lysed cells re-suspended in 50 μl transposase reaction mix, containing 10 mM Tris pH8.0, 5 mM MgCl2, 10% dimethylformamide and 2.5 μl Tn5 transposon + transposase reagent (TDE1 Illumina). Reactions were incubated at 37°C for 45 min, with shaking at 500 rpm. Following transposition, 150 μl reverse-crosslinking buffer (50 mM Tris-Cl, 1 mM EDTA, 1% SDS, 0.2 M NaCl) was added to reactions, and incubated at 65°C overnight at 1000 rpm before DNA purification, using the MinElute PCR purification kit (Qiagen).

Transposed DNA was amplified by PCR for 5 cycles, using KAPA HiFi HotStart ReadyMix, 1.25 μM Ad1 primer and 1.25 μM Ad2.x indexed primers, as described (71). 5 μl of each reaction was taken for qPCR as described, using KAPA HiFi HotStart ReadyMix and 50X KAPA Low ROX for signal normalisation, to determine the additional number of PCR cycles for library amplification. PCR reactions were purified using the MinElute gel extraction kit (Qiagen) according to the manufacturer's instruction, and fragments between 100–1000 bp in size were selected for sequencing. DNA fragmentation of the library was measured using the Bioanalyzer High Sensitivity DNA assay (Agilent), and library concentration quantified by qPCR using the Illumina Library Quantification kit (KAPA). DNA from multiple libraries was pooled at equimolar concentrations to a final concentration of 4 nM. Pooled libraries were sequenced on a HiSeq 3000 (Illumina) using 75 bp paired-end reads. Raw reads were filtered for base quality using FastQC with default parameters and adapters sequences were removed with Trim Galore! Reads passing quality filtering criteria were aligned to GRCm38 using BWA-MEM with default settings (58)

### FRET

HEK293T cells were plated into 24-well plates at 5.5 × 10^4^ cells/well and transfected 24 hours later with 730 ng of each vector encoding mTagBFP and mNeonGreen tagged proteins using polyethyleneimine. Fluorescence signals were recorded 32 hours after transfection with a FACSymphony flow cytometer (BD Biosciences). The FRET signal was recorded in the 405 nm laser line using a 530/30 bandpass filter. DFRET efficiency was calculated as described (72).

### FLIM-FRET

HEK293T cells were plated into 8-well glass coverslip bottom slides (Ibidi) at 2.7 × 10^4^ cells/well. The cells in each well were transfected 24 hours later with 760 ng of each vector as above. The cells were washed with PBS 36 hrs after transfection, fixed with 2% formaldehyde for 15 min, washed twice with PBS and left covered in PBS for image acquisition.

Time-domain fluorescence lifetime images were acquired via time correlated single photon counting (TCSPC) on an Open FLIM microscope (73) at a resolution of 256 by 256 pixels, with 256 time bins and 100 frames accumulated over 300 seconds, via excitation and emission filters suitable for the detection of mTagBFP fluorescence (Excitation filter: Semrock FF01-400/40–25; Beam Splitter: Edmund 48NT-392 30R/70T; Emission filter: Semrock FF01-470/22–25). For each sample a ‘donor’ control image and a ‘donor with acceptor’ image was acquired. FLIM analysis was performed with TRI2 software (v3.0.1.15) using mono-exponential Levenberg-Marquardt fitting (74). The FRET efficiency (FRETeff) for each cell sample region of interest was calculated according to the equation FRETeff = 1–(tDA/tD), where tD is the average lifetime of mTagBFP in the absence of a FRET acceptor from the donor image and tDA is the average lifetime of mTagBFP in the presence of the acceptor mNeonGreen (mNG), from the ‘donor with acceptor’ image.

### Co-immunoprecipitation and immunoblot analysis

HEK293T cells were transiently transfected with expression vectors for mNeonGreen-tagged or HA-tagged proteins using polyethyleneimine. Cells were lysed in lysis buffer (50 mM Tris pH 7.5, 300 mM NaCl, 0.5% IGEPAL CA-630, 1 mM EGTA, 2 mM MgCl_2_) supplemented with 250 U/ml benzonase (Santa Cruz), 1 mM phenylmethylsulfonyl fluoride, 1 mM DTT and 1X complete EDTA-free protease inhibitor (Roche). The cell lysates were sonicated for 10 sec using a Bioruptor Pico (Diagenode) and then incubated on an over-head shaker for 30 min at 4°C. Insoluble material was removed by centrifugation (17,000xg for 10 min), diluted in an equal volume of lysis buffer without NaCl and incubated with 10 μl mNeonGreen-Trap magnetic agarose beads (ChromoTek) on an over-head shaker for 1 hr at 4°C. Beads were washed 2x with 200 μl lysis buffer containing 150 mM NaCl and 4x with lysis buffer containing 500 mM NaCl, resuspended in 1x Laemmli buffer and incubated at 95°C for 9 min. Proteins were resolved on a 12% Tris-glycine gel and transferred to a nitrocellulose membrane. Membranes were probed with anti-β-Tubulin (ab6046, Abcam), anti-HA (3F10, Roche), anti-FLAG (M2, Sigma), anti-GATA3 (HG3-31 (Santa Cruz), anti-T-bet (4B10, eBioscience), anti-β-actin (4967S, Cell Signaling Technologies), and anti-mNeonGreen (#53061, Cell Signaling Technologies).

### Motif analysis

*De novo* and known DNA binding motifs enriched in the sets of gain, loss and invariant GATA3 binding sites were identified with the findMotifsGenome.pl program from the HOMER motif discovery library (75) using the following parameters: mm10 –size 200 –mask. The occurrences of T-box and GATA motifs were quantified using the FIMO motif scanning tool from the MEME suite using a p-value < 0.0004 cutoff (76).

### Oligo-Flow

Oligo-Flow was performed as described (77). We designed DNA oligonucleotides that incorporated the synthetic T-box motif and a control oligo corresponding to the DNA sequence ∼7 kb upstream of *Il5* that lacks a T-box motif:

T-box sense:

5′-TTA GCT AGT CGG CGC TAT AGT TTT CAC ACC TAC GTA ACT GCT GCT AGC TAG TAC-3′

T-box antisense:

5′-GTA CTA GCT AGC AGC AGT TAC GTA GGT GTG AAA ACT ATA GCG CCG ACT AGC TAA-3′

*Il5* sense:

5′-TAC TGA TCT GTA GCA CAT TAA AGG AGA TAG AGG GCT TAG GGC ACA GGG GGT AAA-3′

*Il5* antisense:

5′-TTT ACC CCC TGT GCC CTA AGC CCT CTA TCT CCT TTA ATG TGC TAC AGA TCA GTA-3′

The biotinylated sense oligos and complementary unmodified antisense oligos were annealed in annealing buffer (10 mM Tris-HCl pH 8.0, 50 mM NaCl, 10 mM MgCl_2_ and 1 mM DTT) and incubated with 12 μl streptavidin-coated paramagnetic microspheres (7.5 – 8.5 μm COMPEL, Bangs Laboratories) for 1 hr at RT with rotation. Oligo/bead complexes were washed once in protein binding buffer (10 mM Tris pH 8.0, 100 mM KCl, 10% glycerol, 1 mM EDTA, 1 mM DTT and 160 μg / ml BSA) and resuspended in 462 μl protein binding buffer supplemented with 20 μg / ml poly(dI-dC) (Sigma), 1 mM phenylmethylsulfonyl fluoride and 1X complete EDTA-free protease inhibitor (Roche).

HEK293T cells were transiently transfected with expression vectors for mNeonGreen-tagged WT or mutant T-bet DBD using polyethyleneimine. Cells from one 10 cm plate were lysed in 100 μl lysis buffer (20 mM Tris pH 7.5, 150 mM NaCl, 0.5% IGEPAL CA-630, 0.5 mM EGTA, 2 mM MgCl_2_, 1 mM phenylmethylsulfonyl fluoride, 1 mM DTT and 1X complete EDTA-free protease inhibitor (Roche)). Insoluble material was removed by centrifugation (17,000xg for 10 min) and 20 μl of cleared lysate was incubated with oligo/bead complexes on an over-head shaker for 2 hrs at 4°C. Oligo/bead/protein complexes were washed twice with 200 μl protein binding buffer and resuspended in 600 μl protein binding buffer. Fluorescence was recorded on an LSRFortessa X-20 flow cytometer (BD Biosciences). The background signal from the *Il5* sequence oligo was subtracted from the signal from the T-box motif oligo and the signals for the mutant proteins were then normalised to the signal of the WT T-bet sample.

### Expression constructs

FLAG-T-bet was inserted into BamHI and EcoRI sites of TtTMPV-Neo (Addgene plasmid #27993) to generate TRE-T-bet. The cDNA clones of the Tbx21 Y525F and R1634/R164A mutants were kindly provided by Eun Sook Hwang and Amy Weinmann, respectively, and cloned into pMY to generate FLAG-Tbx21 Y525F-T2A-BSD-IRES-EGFP and FLAG-Tbx21 R163/R164A-T2A-BSD-IRES-EGFP, respectively. Other T-bet and GATA3 mutations were generated via standard two-step overlap PCR mutagenesis using primers containing modified codon sequences. GATA3 R352A was cloned into pMY-PAC-FT2A-HA-GATA3 R352A-IRES-mPlum, as described (29).

The coding regions of full-length T-bet, WT or mutant T-bet DBD (aa 135–326) and the GATA3 DBD (aa 259–369) were fused at the N-terminus to an mNeonGreen or mTagBFP tag separated by a flexible glycine linker (GGGGSGGGGS) using standard PCR cloning methods and inserted into the BamHI and SalI sites of the pMY vector. The coding region of the GATA3 DBD (aa 259–369) was fused at the C-terminus to an mTagBFP tag separated by a flexible glycine linker (GGGGSGGGGS) using standard PCR cloning methods and inserted into the BamHI and SalI sites of the pMY vector (29).

To generate N-terminally HA tagged GATA3 constructs the cDNAs of WT GATA3 and C320G, G342S and K346A + H348L mutants were inserted into pcDNA3 downstream of an in-frame double HA-tag. A C-terminally HA-tagged GATA3 construct was generated by inserting the coding region of GATA3 into pcDNA3 upstream of an in frame double HA-tag. The pcDNA3-HA-CyclinT1 construct was described in (57).

### Structural analysis and alignment

Crystal structures of the T-bet DBD (PDB id 5T1J) and TBX5 DBD (PDB id 2 × 6U) were used for the structural analysis. Structural alignment between the two monomeric chains was achieved using the pairwise structural alignment mode on the DALI server (http://ekhidna2.biocenter.helsinki.fi/dali/) and used to identify the T-bet DBD residues corresponding to the TBX5 amino acids previously predicted to be involved in GATA4 binding. Root-mean-square deviation calculations and figures were prepared using Pymol (Schröndinger, LLC).

### Statistical Analyses

Biological replicates are defined as individual cell harvests of the same EL4 cell line on different days and independent transfections of HEK293T cells on different days. Technical replicates for quantitative PCR data represent individual PCR reactions of the same sample and for microscopy data individual cells from the same transfection. Number of technical or biological replicates (n), definitions of center and dispersion are indicated in the figure legends. Statistical significance of a test group versus a reference group was determined using one-tailed Student's t-test assuming normal distribution of each test group. The null hypothesis was rejected at a significance level α < 0.05.

## RESULTS

### T-bet redistributes GATA3 away from its canonical binding sites

We first sought to determine if co-expression of T-bet and GATA3 had an effect on the genome binding profiles of either factor. To address this question, we expressed FLAG-T-bet and HA-GATA3, either individually or together, in the C57BL/6 mouse T lymphoblast cell line EL4 (78) (Supplementary Figure S1A). Expression of T-bet resulted in production of IFNγ by EL4 cells, as observed previously (17), but expression of GATA3 did not lead to production of IL-4 (data not shown). The cells were stimulated with PMA and ionomycin and T-bet and GATA3 binding sites identified across the genome by chromatin immunoprecipitation sequencing (ChIP-seq).

In order to validate the EL4 cell model, we first compared the sites occupied by T-bet and GATA3 when expressed in EL4 cells individually with the sites occupied by these factors in primary T_H_1 cells (62,63) or primary T_H_2 cells (64), respectively (Supplementary Figure S1B-D). This showed that the binding profiles of T-bet and GATA3 were similar between EL4 cells and primary T cells, but that some sites exhibited greater occupancy in one cell type or the other. Analysis of transcription factor binding motifs at these sites revealed enrichment of similar motifs, including strong enrichment of T-box motifs at sites uniquely bound by T-bet in EL4 cells (Supplementary Figure S1E). Similarly, sites uniquely bound by GATA3 in EL4 cells exhibited strong enrichment for GATA motifs (Supplementary Figure S1F). Thus, we conclude that T-bet and GATA3 bind similar sets of sites with similar motifs in EL4 cells compared with primary T cells.

**Figure 1.**
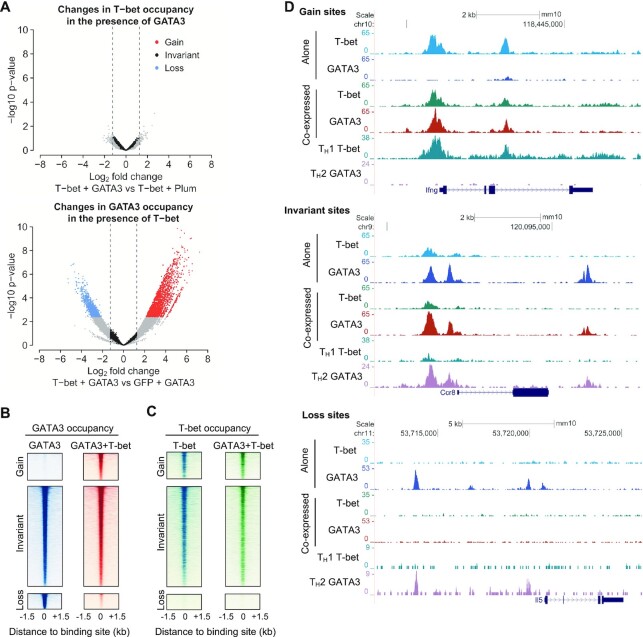
T-bet redistributes GATA3 from its canonical binding sites to T-bet binding sites. (**A**) Top: Changes in T-bet occupancy in cells co-expressing T-bet and GATA3 compared with cells expressing T-bet alone (T-bet + Plum). Each point represents a site bound by T-bet in one of the two cell types. Bottom: Changes in GATA3 occupancy in cells co-expressing T-bet and GATA3 compared with cells expressing GATA3 alone (GFP + GATA3). Each point represents a site bound by GATA3 in one of the two cell types. For both transcription factors, sites with significantly higher occupancy (FDR <= 0.05; gain sites), significantly lower occupancy (FDR <= 0.05; loss sites), or no change in occupancy (FDR > 0.05, -1.25 < log2 FC < 1.25; invariant sites) in the presence of the other factor are coloured as indicated (*n* = 2 biological replicates). (**B**) Heatmaps showing GATA3 occupancy (reads/million) at GATA3 gain, invariant and loss sites defined in A in the presence or absence of T-bet (representative of two biological replicates). (**C**) Heatmap showing T-bet occupancy at GATA3 gain, invariant and loss sites defined in A in the presence or absence of GATA3 (representative of two biological replicates). (**D**) Binding profiles (reads/million) for T-bet and GATA3 at genes associated with GATA3 gain (top), invariant (center) and loss (bottom) sites in EL4 cells expressing each factor alone or co-expressing the two factors (representative of two biological replicates). T-bet occupancy in mouse T_H_1 cells and GATA3 occupancy in mouse T_H_2 cells are shown below for comparison.

We next determined whether co-expression of T-bet and GATA3 had any effect on T-bet genome occupancy. Comparison of all T-bet binding sites between cells expressing T-bet alone and cells co-expressing T-bet and GATA3 did not reveal any significant changes in T-bet binding in the presence of GATA3 (Figure 1A). In contrast, we observed dramatic changes in GATA3 genome occupancy when T-bet was present (Figure 1A and B). Specifically, 7% (988) of GATA3 binding sites were lost in the presence of T-bet and 13% (1,760) of GATA3 binding sites were gained in the presence of T-bet (Figure 1A and B). In addition, around half (6,622) of GATA3 binding sites were invariant (significantly bound by GATA3 in the presence and absence of T-bet). Thus, when both factors were co-expressed, GATA3 had no effect on T-bet localisation while T-bet induced a major redistribution of GATA3 to a different set of sites.

We have previously noted that GATA3 adopts an alternative binding profile in primary human T_H_1 cells versus T_H_2 cells (29). We have also demonstrated that a large proportion of transcription factor binding sites are shared between human and mouse CD4^+^ T cells (70). Thus, to determine if the alternative binding profile adopted by GATA3 in T_H_1 cells could be related to the redistribution of GATA3 by T-bet in EL4 cells, we identified locations in the human genome orthologous to murine GATA3 binding sites using liftOver and plotted GATA3 occupancy at these locations in human T_H_1 and T_H_2 cells (Supplementary Figure S2A). We found that sites at which GATA3 was gained in the presence of T-bet exhibited higher levels of GATA3 occupancy in T_H_1 cells compared to T_H_2 cells. Reciprocally, sites at which GATA3 was lost in the presence of T-bet exhibited lower levels of GATA3 occupancy in T_H_1 cells, while sites at which GATA3 was invariant in the presence or absence of T-bet showed equal GATA3 occupancy in T_H_1 and T_H_2 cells. Thus, these results suggest that the phenomenon of T-bet-mediated GATA3 redistribution occurs in both EL4 cells and primary T cells.

We considered two scenarios by which T-bet could bring about redistribution of GATA3. Firstly, T-bet could compete with GATA3 for its binding sites, evicting it and forcing GATA3 to redistribute to alternative locations. Alternatively, T-bet could induce GATA3 to localise to its own binding sites, thereby depleting GATA3 from its canonical binding sites. To distinguish between these possibilities, we measured T-bet binding at sites at which GATA3 was lost, gained or invariant in the presence of T-bet (Figure 1C). This showed that T-bet was absent from sites at which GATA3 was lost, which rules out an eviction mechanism. In contrast, T-bet was bound with GATA3 at sites to which GATA3 was invariant or gained in the presence of T-bet. This suggests that T-bet recruits GATA3 to its own binding sites. These binding patterns could also be observed at individual genes (Figure 1D and Supplementary Figure S2B). For example, when expressed alone, GATA3 occupied sites at *Ccr3* but these disappeared in the presence of T-bet. In contrast, GATA3 did not bind to *Ifng* when expressed alone, but co-occupied the gene with T-bet when the two factors were co-expressed. Thus, when both factors are present, T-bet induces redistribution of GATA3 from its canonical binding sites to T-bet binding sites.

### The extent of GATA3 redistribution is dependent on the relative levels of T-bet and GATA3

**Figure 2.**
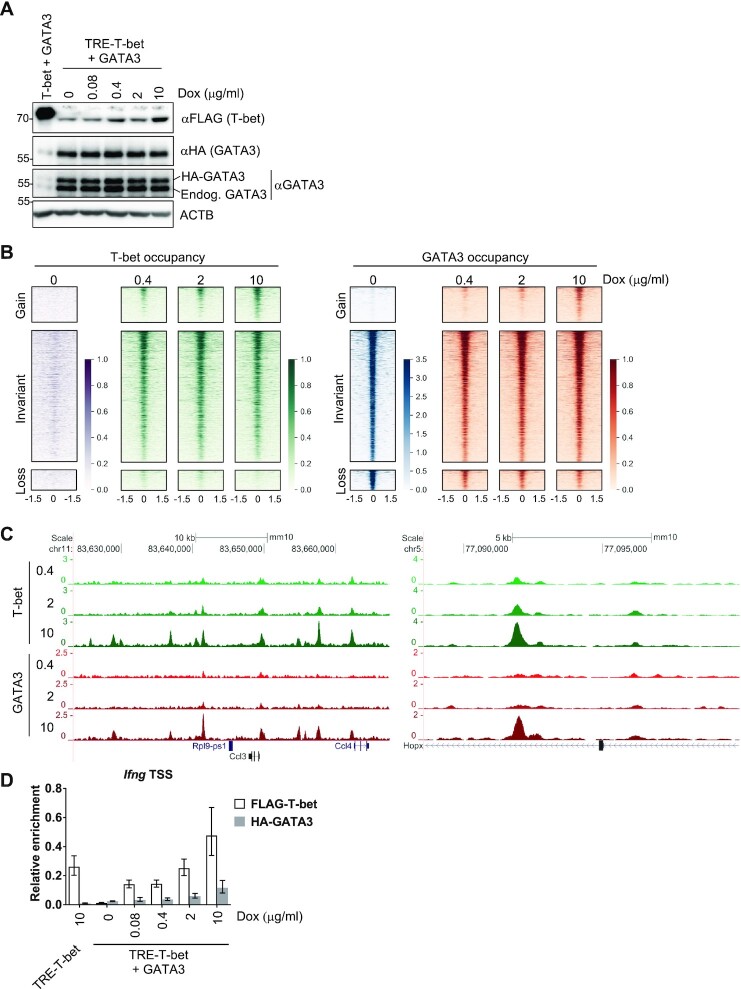
The extent of GATA3 redistribution is dependent on the relative levels of T-bet and GATA3. (**A**) Immunoblot with αFLAG, αHA, αGATA3 and αACTB in EL4 cells constitutively expressing FLAG-T-bet and HA-GATA3 (used in Figure 1) and EL4 cells that constitutively express HA-GATA3 and a dox-inducible FLAG-T-bet construct (TRE-T-bet) after treatment with 0–10 μg/ml dox for 48 hours. The positions of endogenous GATA3 and HA-GATA3 in the αGATA3 blot are labelled. Representative of two biological replicates. (**B**) Heatmaps showing T-bet and GATA3 occupancy (reads/million, according to the scales to the right) at GATA3 gain, invariant and loss sites defined in Figure 1A after treatment of TRE-T-bet + GATA3 EL4 cells with 0.4, 2 or 10 μg/ml dox for 48 hours (one replicate). T-bet and GATA3 occupancy in GATA3 + GFP cells (used in Figure 1, not treated with dox) are shown at the same sites for comparison. (**C**) Binding profiles (reads/million) for T-bet and GATA3 at genes associated with GATA3 gain sites in cells described in B (one replicate). (**D**) Enrichment of *Ifng* promoter DNA relative to input DNA by ChIP for FLAG-T-bet (white) or HA-GATA3 (grey) in cells shown in A., measured by qPCR (mean and SD, *n* = 3 technical replicates of one biological replicate).

We considered whether the relative levels of T-bet and GATA3 might affect the ability of T-bet to redistribute GATA3. To test this, we generated an EL4 cell line that expressed higher levels of HA-GATA3 and a doxycycline (dox)-inducible FLAG-T-bet construct through which the T-bet expression level could be controlled by titration of dox (Figure 2A). These cells contained higher levels of GATA3 and lower levels of T-bet, which were more comparable to primary T_H_ cells (compare Figure 2A with Supplementary Figure S1A). To determine the effect varying the level of T-bet expression on GATA3 occupancy, we treated the cells with 0.4, 2 or 10 mg/ml dox for 48 hours and then performed ChIP-seq for HA-GATA3 and FLAG-T-bet. We also compared these data with GATA3 occupancy in the absence of T-bet in our previously generated GATA3 + GFP cell line. We found that regardless of the level of T-bet expression, T-bet recruited GATA3 to T-bet-dependent gain sites and the greater the level of T-bet expression, the greater the level of GATA3 recruitment to these positions (Figure 2B). GATA3 recruitment to T-bet target sites in proportion to the level of T-bet occupancy was also apparent at individual genes (Figure 2C) and was also confirmed at *Ifng* by ChIP-qPCR (Figure 2D). We also found that the greater the expression of T-bet, the greater the depletion of GATA3 from loss sites (Figure 2B), although the lower level of T-bet expression in these cells was not sufficient to completely remove GATA3 from these sites. These results demonstrate that the greater the amount of T-bet relative to GATA3, the greater the degree of GATA3 redistribution.

There was also evidence of T-bet recruitment to GATA3 loss sites when present at its lowest level in the 0.4 mg/ml dox-treated sample (Figure 2B), suggesting that GATA3 may be able to recruit T-bet to GATA3 binding sites when GATA3 is in excess. However, the level of T-bet recruitment to GATA3 binding sites was low compared to the level of GATA3 recruitment to T-bet-dependent sites. These data suggest that T-bet and GATA3 are both able to recruit the other factor to their binding sites, but that the effect of T-bet on GATA3 is dominant, consistent with the previously observed redistribution of GATA3 to T-bet binding sites in human T_H_1 cells (29).

### T-bet-mediated GATA3 redistribution is associated with silencing of the T_H_2 gene expression program

We next sought to determine whether the redistribution of GATA3 in the presence of T-bet was associated with changes in gene expression. To address this, we performed RNA-seq in the T-bet-expressing, GATA3-expressing, and T-bet and GATA3 co-expressing cells that we used for the initial transcription factor binding studies. Using gene set enrichment analysis (GSEA) we found, as expected, that expression of T-bet alone upregulated T_H_1-specific genes and downregulated T_H_2-specific genes (Supplementary Figure S3A). Correspondingly, expression of GATA3 alone had the opposite effect, upregulating T_H_2 genes and downregulating T_H_1 genes (Supplementary Figure S3B). Thus, in isolation each LD-TF can establish its canonical lineage-specific gene expression program in EL4 cells.

**Figure 3.**
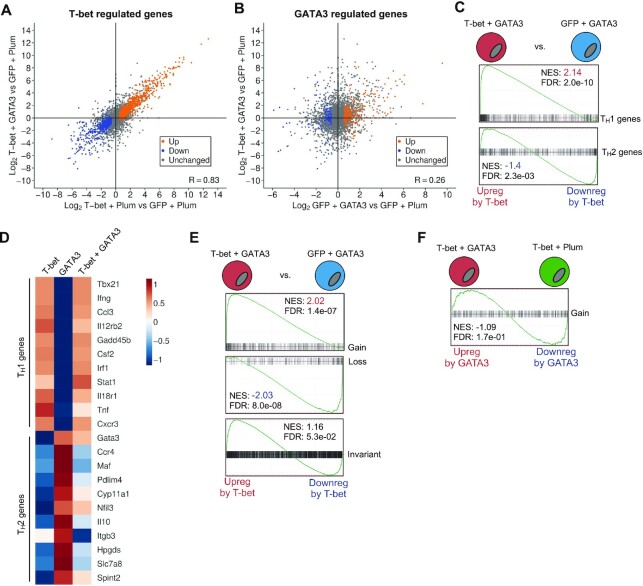
T-bet-mediated GATA3 redistribution is associated with silencing of the T_H_2 gene expression program. (**A**) Log2 FC in gene expression in cells expressing T-bet and Plum versus GFP and Plum plotted against log2 FC in gene expression in cells expressing T-bet and GATA3 versus GFP and Plum (*n* = 3 biological replicates). Genes up- or down-regulated (FDR <= 0.05, log2 FC >|1.5|) in cells expressing T-bet and Plum versus GFP and Plum are labelled. T-bet induces similar changes in gene expression in the presence or absence of GATA3 (r = 0.83). (**B**) Log2 FC in gene expression between cells expressing GFP and GATA versus GFP and Plum plotted against log2 FC in gene expression between cells expressing T-bet and GATA3 versus GFP and Plum (*n* = 3 biological replicates). Genes up- or down-regulated in cells expressing GFP and GATA3 versus GFP and Plum are labelled. The changes in gene expression vary depending on whether T-bet is present (r = 0.26). (**C**) Gene set enrichment analysis (GSEA) of T_H_1 and T_H_2 gene signatures compared with differences in gene expression between cells expressing T-bet and GATA3 versus GFP and GATA3. NES, normalized enrichment score; a positive value indicates enrichment of the gene signature in the set of up-regulated genes and *vice versa*. FDR, false discovery rate. (**D**) Heatmap of gene expression changes (z-scores; scale on the right) for selected T_H_1 and T_H_2 signature genes in T-bet + Plum, GFP + GATA3 and T-bet + GATA3 cells relative to GFP + Plum cells (*n* = 3 biological replicates). (**E**) GSEA of genes associated with GATA3 gain, loss or invariant sites compared with differences in gene expression between cells expressing T-bet and GATA3 versus cells expressing GFP and GATA3. Details as for C. (**F**) GSEA of genes associated with GATA3 gain sites compared with differences in gene expression between cells expressing T-bet and GATA3 versus T-bet and Plum. Details as for C.

We then determined the effect of T-bet on gene expression when co-expressed with GATA3. Comparing the gene expression changes induced by T-bet alone with those induced by T-bet and GATA3, we found that T-bet established essentially the same expression profile in the presence and absence of GATA3 (r = 0.83, p < 2.2e-16; Figure 3A). In contrast, there was no correlation between the expression profile of T-bet / GATA3 co-expressing cells and cells containing GATA3 alone (r = 0.26, p < 2.2e-16), demonstrating that T-bet prevents GATA3 from establishing its expression programme (Figure 3B). As for cells containing T-bet alone, cells expressing both T-bet and GATA3 upregulated T_H_1-specific genes in the absence of significant upregulation of T_H_2 genes (Figure 3C and D). Thus, when both factors are expressed together, T-bet function dominates, with cells adopting a T_H_1 expression profile, characterised by induction of T_H_1 genes and repression of T_H_2 genes compared to cells expressing GATA3 alone.

We next asked how these changes in gene expression were related to GATA3 redistribution. We tested whether genes associated with sites at which GATA3 was lost, gained or invariant in the presence of T-bet were enriched among the sets of genes up- or down-regulated in cells co-expressing T-bet and GATA3 versus cells expressing GATA3 alone. This analysis revealed that genes downregulated in the presence of T-bet were enriched for sites at which GATA3 was lost and that upregulated genes were enriched for sites at which GATA3 was gained (Figure 3E). In contrast, GATA3 invariant sites were not significantly enriched among either upregulated or downregulated genes (Figure 3E).

We also asked whether the recruitment of GATA3 to T-bet binding sites had any effect on the expression of the associated genes. Comparing gene expression between cells co-expressing T-bet and GATA3 with cells expressing T-bet alone, we found no enrichment of sites to which GATA3 was gained at the genes that were up- or down-regulated in the presence of GATA3 (Figure 3F), suggesting that GATA3 recruitment had no effect on the expression of T-bet target genes. Thus, we conclude that T-bet-mediated GATA3 redistribution results in suppression of the T_H_2 gene expression program without impacting T_H_1 gene expression.

### GATA3 removal depletes H3K27ac from its binding sites

**Figure 4.**
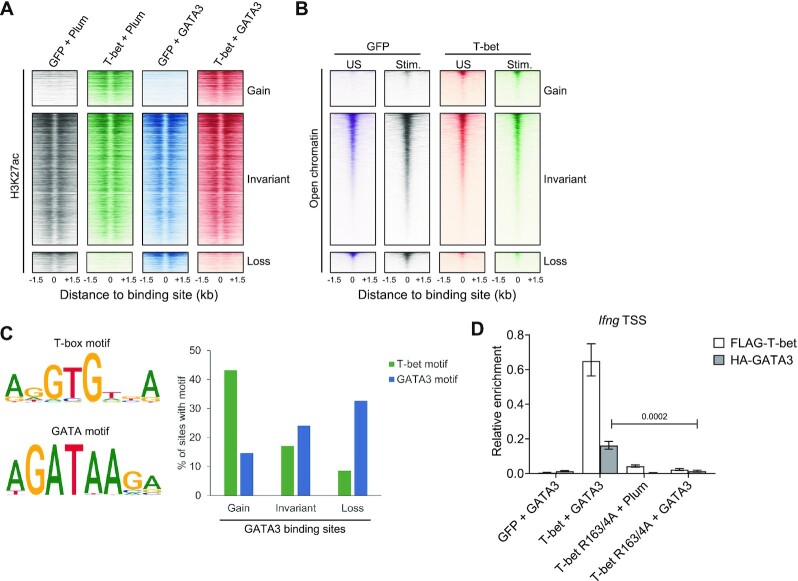
T-bet recruits GATA3 to sites of open chromatin in a manner dependent on T-bet DNA binding. (**A**) Heatmaps showing H3K27ac enrichment at GATA3 gain, invariant and loss sites in cells expressing GFP and Plum, T-bet and Plum, GFP and GATA3 or GATA3 and T-bet (one biological replicate). (**B**) Heatmaps showing chromatin accessibility (measured by ATAC-seq) at GATA3 gain, invariant and loss sites in unstimulated (US) and stimulated (Stim.) cells expressing GFP or T-bet (representative of two biological replicates). (**C**) Left: T-box and GATA DNA binding motifs enriched at T-bet binding sites and GATA3 binding sites, respectively, in cells expressing either factor alone. Right: Proportion of GATA3 gain, invariant and loss sites that contain the T-box or GATA3 DNA binding motifs. (**D**) Enrichment of *Ifng* promoter DNA relative to input DNA by ChIP for FLAG-T-bet (white) or HA-GATA3 (grey) in EL4 cells expressing GFP and GATA3, WT T-bet and GATA3, T-bet DNA binding mutant R163A/R164A and Plum or T-bet R163A/R164A and GATA3 measured by qPCR (mean and SD, *n* = 3 technical replicates of one biological replicate, one-tailed Student's t-test).

Our results show that T-bet removes GATA3 from a subset of its binding sites and that this is associated with gene repression. We sought to determine whether this repressive effect of T-bet was reflected in changes to chromatin at GATA3 binding sites by profiling the active histone modification H3K27ac by ChIP-seq in the presence or absence of T-bet and GATA3. We found increased levels of H3K27ac at sites to which GATA3 was recruited in the presence of T-bet (Figure 4A), consistent with the positive effect of T-bet on the expression of genes to which GATA3 was recruited. In contrast, the presence or absence of GATA3 had no effect on H3K27ac at these GATA3 gain sites (Figure 4A), consistent with the lack of effect of GATA3 on the expression of these genes (Figure 3F). Reciprocally, we found decreased levels of H3K27ac at sites from which GATA3 was lost in the presence T-bet (Figure 4A), consistent with the reduced expression of these genes in the presence of T-bet. We conclude that T-bet-mediated GATA3 redistribution is associated with changes in chromatin modification at GATA3 binding sites that is consistent with silencing of the T_H_2 gene expression program.

### GATA3 redistribution to new sites requires T-bet DNA binding activity

We considered that T-bet may recruit GATA3 to new binding sites by opening chromatin and making the sites accessible. To test this, we performed ATAC-seq in unstimulated and PMA + ionomycin stimulated EL4 cells in the presence and absence of T-bet. We found that GATA3 loss sites and GATA3 invariant sites tended to be accessible in the absence of T-bet and that this accessibility increased upon cell stimulation (Figure 4B). In the presence of T-bet, chromatin accessibility was reduced at GATA3 loss and GATA3 invariant sites, consistent with the repressive effect of T-bet on the genes associated with these sites. In contrast, GATA3 gain sites tended to be inaccessible in the absence of T-bet and exhibited increased chromatin accessibility when T-bet was present independently of the cellular activation status. Thus, this ATAC-seq analysis indicates that T-bet may recruit GATA3 to gain sites by opening chromatin and thereby allowing GATA3 to access otherwise inaccessible DNA sequence motifs.

To determine whether GATA3 could be directly binding to DNA at gain sites, we identified T-box and GATA binding motifs significantly enriched at their binding sites in cells expressing T-bet or GATA3 alone, respectively, and quantified the occurrences of these motifs at GATA3 gain, loss and invariant sites (Figure 4C). We found that a GATA motif was present at 32% of loss sites but at only 14% of gain sites. We found the opposite pattern for the T-box motif, with 43% of gain sites containing the motif compared with only 8% of loss sites (Figure 4C). These data suggest that it is T-bet DNA binding motifs rather than GATA DNA binding motifs that are important for GATA3 redistribution.

To determine if additional factors may have a role in changes in GATA3 occupancy in the presence of T-bet, we systematically identified motifs enriched at GATA3 gain, loss and invariant sites (Supplementary Figure S4A). In comparison to GATA3 invariant sites, we found that T-bet-dependent GATA3 gain sites exhibited strong enrichment for T-box motifs. No other motif type exhibited this relative enrichment at GATA3 gain sites, consistent with a model in which T-bet directly recruits GATA3 to these sites. Comparison of GATA3 invariant sites to GATA3 loss sites showed no differences in motif enrichment, consistent with the model that GATA3 loss is caused by sequestration by T-bet rather than changes in binding of a third factor to these sites (Supplementary Figure S4B). We also looked for enrichment of potential composite T-bet and GATA3 DNA binding motifs at GATA3 gain sites through which T-bet and GATA3 may bind together but could not find any evidence of such motifs (Supplemental Figure S4C).

The low frequency of GATA motifs coupled with the high frequency of T-box motifs at T-bet-dependent GATA3 binding sites indicated a requirement for T-bet DNA binding for GATA3 recruitment to these sites. To test this, we measured GATA3 recruitment in the presence of the T-bet DNA-binding mutant R163A + R164A (79). ChIP for HA-GATA3 showed that mutation of the T-bet DNA binding domain (DBD) abrogated its ability to recruit GATA3 to *Ifng* (p = 0.0002, Student's t-test, Figure 4D). Thus, we conclude that recruitment of GATA3 to new sites requires T-bet DNA binding.

### Recruitment of GATA3 to T-bet binding sites does not require its canonical DNA binding activity

Given that GATA3 was recruited to sites enriched for the T-box motif in a manner that required T-bet DNA binding, we considered the possibility that GATA3 was bound to these sites via interaction with T-bet. In support of such a model, two independent studies have reported that T-bet and GATA3 interact in co-IP experiments (23,80). T-bet has been reported to interact with GATA3 via its C-terminus, with mutation of Y525 to phenylalanine disrupting this interaction (23). However, we found that T-bet Y525F still interacted with GATA3 (Supplementary Figure S5A) and, consistently, could still recruit GATA3 to *Ifng* (Supplementary Figure S5B).

**Figure 5.**
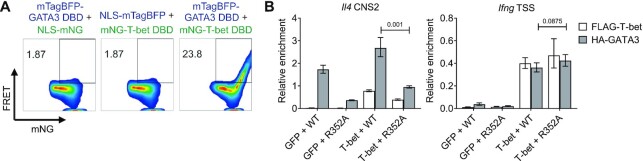
Recruitment of GATA3 to T-bet binding sites does not require its canonical DNA binding activity. (**A**) Flow cytometric measurement of FRET between mTagBFP-GATA3 DBD and mNG-NLS, mTagBFP and mNG-T-bet DBD or mTagBFP-GATA3 DBD and mNG-T-bet DBD. mTag-BFP was excited with the violet laser and fluorescence emission from mNG measured in the same laser line with a 530/30 bandpass filter. The proportion of FRET-positive cells are indicated for each combination of constructs. Representative of two biological replicates. (**B**) Enrichment of *Il4* CNS2 and *Ifng* promoter DNA relative to input DNA by ChIP for anti-FLAG (white) or anti-HA (grey) in EL4 cells expressing WT GATA3 or GATA3 R352A DNA binding mutant in the absence (GFP) or presence of T-bet (mean and SD, *n* = 3 technical replicates of one biological replicate, one-tailed Student's t-test).

The T-box family member TBX5 and the GATA family member GATA4 interact in cardiomyocytes (81). Mutations in the TBX5 and GATA4 DBDs disrupt the interaction between the proteins suggesting that interactions between T-box and GATA factors may be mediated by their DBDs (81). To test whether T-bet and GATA3 interacted via their DBDs, we co-expressed mTagBFP-tagged GATA3 DBD and mNeonGreen-tagged T-bet DBD in HEK293T cells and measured Förster resonance energy transfer (FRET) between the fluorescent proteins by flow cytometry. We identified a strong FRET signal in the presence of fluorescent T-bet and GATA3 that was absent if either protein was replaced with the fluorophore alone (Figure 5A). Thus, these results support a model in which T-bet recruits GATA3 to its binding sites through direct interaction between their DBDs.

Our findings thus far are compatible with two functionally different models of GATA3 binding to gain sites; either T-bet-mediated chromatin opening allows GATA3 to bind to its canonical GATA motif at these sites or that T-bet binds GATA3 and brings it to T-bet binding sites without the requirement for GATA3 DNA binding function. To distinguish between these models, we measured the recruitment of a previously identified GATA3 DNA binding mutant (R352A) (82) to T-bet-dependent binding sites in the absence and presence of T-bet. We found that while the R352A mutation reduced GATA3 binding to a canonical GATA-containing site at *Il4* CNS2, it had no effect on T-bet mediated GATA3 recruitment to *Ifng* (Figure 5B). Thus, together with our T-bet DNA binding mutant ChIP data (Figure 4D), these results demonstrate that T-bet DNA binding is required for T-bet-mediated GATA3 recruitment while GATA3 DNA binding activity is dispensable. This suggests that GATA3 does not directly bind DNA at T-bet-dependent binding sites and is instead associated with these sites through interaction with T-bet.

### DNA binding and GATA3 interaction are tightly linked aspects of T-bet function

**Figure 6.**
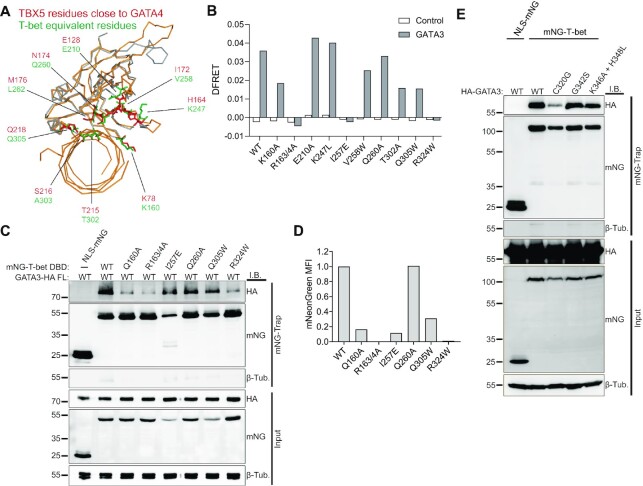
T-bet residues required for interaction with GATA3 are also required for DNA binding. (**A**) Structural alignment of the mouse T-bet DBD (PDB ID 5T1J; grey) and DNA-bound human TBX5 DBD (PDB ID 2 × 6U; orange) crystal structures. TBX5 residues in close proximity to GATA3 according to molecular docking of the TBX5 and GATA3 crystal structures (81) are highlighted in red and the equivalent T-bet residues are in green. (**B**) FRET efficiency (DFRET; relative to mNG-T-bet and mTagBFP) measuring interaction between WT or mutant mNG-T-bet DBD and mTagBFP-GATA3 DBD in HEK293T cells measured by flow cytometry (representative of two biological replicates). (**C**) Immunoblots of HA-GATA3, mNG and β-tubulin in input samples and mNG immunoprecipitates from HEK293T cells expressing full-length (FL) GATA3-HA with NLS-mNG or WT or mutant mNG-T-bet DBD (representative of two biological replicates). (**D**) Binding of mNG-tagged WT and mutant T-bet DBD to biotinylated T-box motif-containing DNA oligos measured by flow cytometry. For each protein, the mean fluorescence intensity (MFI) from non-specific binding to DNA oligos lacking T-box motifs was subtracted. Representative of two biological replicates. (**E**) Immunoblots of HA-GATA3, mNG and β-tubulin in input samples and mNG immunoprecipitates from HEK293T cells expressing WT or mutant HA-GATA3 with NLS-mNG or mNG-T-bet. Representative of two biological replicates.

Given that the T-bet DBD is sufficient for interaction with GATA3, we considered how T-bet's GATA3 binding activity was related to its DNA binding activity. The two residues in TBX5 (G80R and R237W/Q) previously shown to be required for interaction with GATA4 (81) are also required for TBX5 DNA binding (81,83,84), indicating the two activities may be tightly linked. To understand the relationship between T-bet's GATA3 and DNA binding activities in more detail, we utilised a previously performed *in silico* docking of the crystal structures of the TBX5 DBD and the GATA3 DBD (85). This analysis identified twelve amino acids in TBX5 that were located at the GATA3 DBD interaction surface. Using sequence and structural homology search against a murine T-bet DBD structure (86), we mapped ten of these amino acids to positions within T-bet, while we were unable to find equivalent residues for two others (TBX5 A130 and M131) (Figure 6A). Notably, the ten residues that could be mapped to murine T-bet all cluster together in an area close to the DNA below the dimerization interface (in the crystal structure T-bet forms a dimer that bridges two DNA molecules (86)).

Based on the hypothesis that these ten residues would contribute to the interaction of T-bet with GATA3, we generated point mutations to test their effect on GATA3 interaction. We excluded three amino acids because they were either known to contact DNA phosphates (S261) or were located next to amino acids that do so (L262 and A303) (86). Furthermore, we included two additional residues in this analysis: I257, because of its location within the same region predicted to contact GATA3, and R324, equivalent to R237 in TBX5, because of its importance for both DNA binding and interaction with GATA4 (81,83,84). To determine the contribution of these residues to the interaction of T-bet with GATA3, we generated mNeonGreen (mNG)-tagged mutant T-bet proteins by changing the WT residues to amino acids of altered electrical charge, polarity, hydrophilicity or size. Additionally, we included the known T-bet DNA-binding mutant R163A/R164A (79) to assess the importance of T-bet DNA binding activity for its interaction with GATA3. Flow-cytometric FRET measurements of HEK293T cells transfected with mTagBFP-tagged GATA3-DBD and mNG-tagged WT or mutant T-bet DBD revealed a ∼50% reduction in the interaction of T-bet Q160A, T302A and Q305W with GATA3 while I257E completely abrogated interaction with GATA3 (Figure 6B and Supplementary Figure S6A). The inability of the I257E mutant to interact with GATA3 was confirmed by fluorescence lifetime imaging microscopy (FLIM)-FRET (Supplementary Figure S6B). In addition, the R163A/R164A and R324W mutations also completely abrogated interaction with GATA3, indicating that residues required for DNA binding are also involved in the interaction with GATA3.

To confirm that the reduction in FRET activity reflected reduced interaction between T-bet and GATA3, we also performed co-IP experiments with mNG-T-bet DBD and HA-tagged GATA3 in cell lysates that were treated with benzonase to degrade DNA that could potentially mediate indirect interactions between the proteins (Figure 6C). These experiments confirmed that the interaction of the DNA-binding mutants R163/4A and R324W with GATA3 were diminished compared to the WT T-bet DBD, with T-bet Q160A, I257E and Q305W also exhibiting reduced GATA3 interaction.

These results demonstrate that residues in the T-bet DNA binding domain are required for interaction with GATA3. Thus, we next determined the effect of these mutations on T-bet DNA binding activity using our previously developed *in vitro* OligoFlow assay (77). Biotinylated DNA oligos incorporating a synthetic T-box motif were conjugated to streptavidin-coated polystyrene beads, incubated with cell lysates from HEK293T cells transfected with mNG-tagged T-bet and fluorescence intensity measured by flow cytometry (Figure 6D). This revealed that all the mutants that displayed reduced interaction with GATA3 also showed a corresponding reduction in DNA binding activity. We conclude that T-bet residues required for interaction with GATA3 are also required for DNA binding, indicating that T-bet's GATA3 binding and DNA binding activities are tightly linked.

We next asked whether GATA3′s T-bet binding and DNA binding activities were also tightly linked in the same way. We selected four GATA4 mutants [C273G, G296S and K299A + H301L (81,87,88)] that were previously shown to impair both DNA binding and interaction with TBX5 and identified the equivalent residues in GATA3 (C320G, G342S, K346A + H348L) by aligning the primary amino acid sequence of the two proteins. Co-IP of mNG-tagged T-bet and HA-GATA3 in HEK293T cells showed that G342S and K346A + H348L maintained interaction with T-bet but that C320G showed a strongly reduced interaction (Figure 6E). We then assessed the effect of the C320G mutation on GATA3 DNA binding by ChIP. We found that occupancy of HA-GATA3 C320G at the *Il4* locus in HEK293T cells was strongly reduced compared to that of WT HA-GATA3 (Supplementary Figure S6C). These data indicate that GATA3 interacts with T-bet through a subset of the residues that mediate its interaction with DNA and, taken together with the results from the T-bet mutants, this is consistent with a model in which the interaction between T-bet and GATA3 is mediated by their DNA binding surfaces.

## DISCUSSION

**Figure 7.**
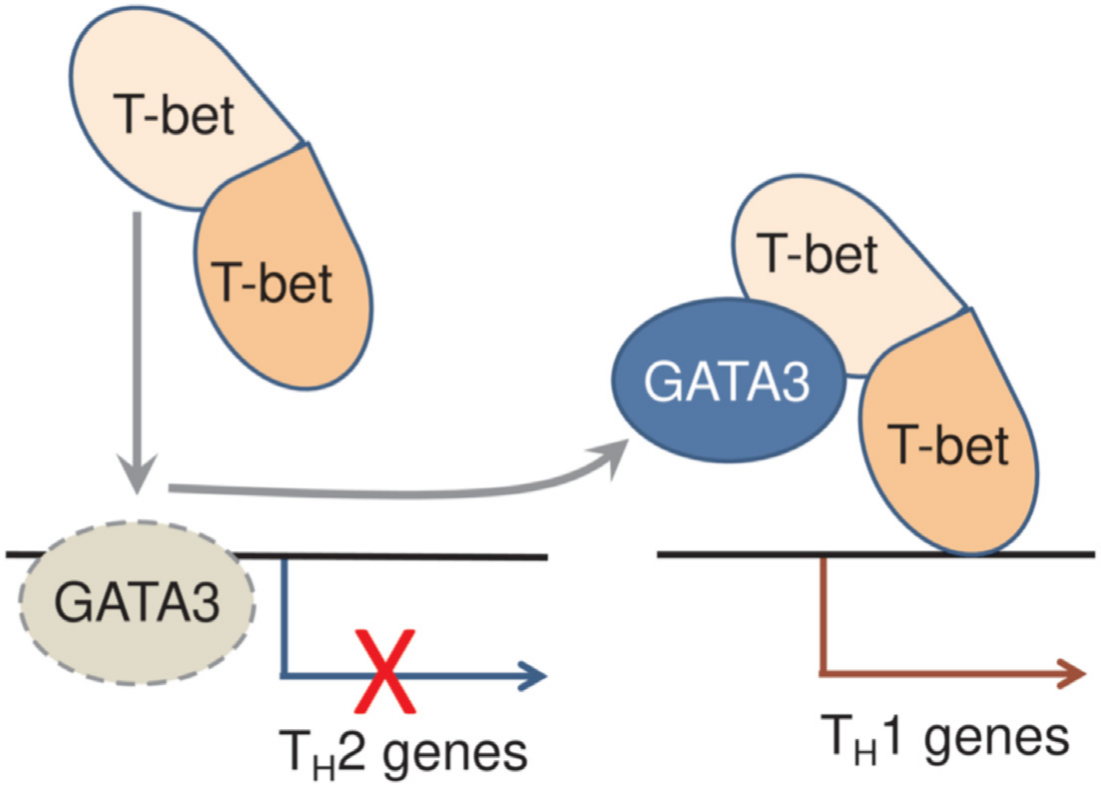
Model of T-bet-mediated GATA3 redistribution from T_H_2 genes to T_H_1 genes. In the absence of T-bet, T_H_2 genes are bound and activated by GATA3. When both factors are co-expressed, the T-bet DBD interacts with the GATA3 DBD, preventing it from binding to its canonical GATA sites, and redistributes GATA3 to T-bet binding sites at T_H_1 genes. This results in the silencing of the T_H_2 gene expression program and activation of the T_H_1 gene expression program. T-bet dimerization could allow it to interact with both GATA3 and its target sites on DNA.

LD-TFs that drive mutually exclusive cell differentiation programs are frequently co-expressed in cells but the impact of this on transcription factor function and cell state are unclear. Using co-expression of the T_H_1 and T_H_2 cell LD-TFs T-bet and GATA3 as a model, we have found that T-bet directly interacts with GATA3, redistributing it from its canonical sites at T_H_2 genes to a new set of binding sites at T_H_1 genes, driving T_H_1 gene expression while silencing the T_H_2 gene expression program (Figure 7).

The mechanism of T-bet-mediated GATA3 redistribution that we have defined here likely has important consequences for the regulation of CD4^+^ T cell differentiation. GATA3 adopts an alternative binding profile in primary human T_H_1 cells, which is characterised by depletion of GATA3 from T_H_2 genes and gain at T-bet binding sites that lack GATA motifs at T_H_1 genes (29). Thus, despite the differences between the cell types, the GATA3 redistribution mechanism that we describe in EL4 cells also appears to operate in primary T cells. However, directly testing the importance of T-bet-mediated GATA3 redistribution to T_H_1 and T_H_2 differentiation *in vivo* is complicated by our discovery that the same residues are required for T-bet's GATA3 interaction and DNA binding functions, which therefore cannot be separated.

The question arises as to the advantage of the GATA3 redistribution mechanism over other possible means by which the T_H_2 gene expression program could be repressed. Although T-bet is a transactivator, T-bet-mediated GATA3 redistribution allows T-bet to mediate both activation of T_H_1 genes and the repression of T_H_2 genes. The ability of T-bet to repress GATA3 function in this way likely relates to the addition role of GATA3 in T cell maturation, the outcome of which is that GATA3 is already present in cells at the time that T-bet becomes expressed. Repressing T_H_2 genes by redistributing GATA3 rather than silencing *Gata3* expression completely may also provide a degree of plasticity in which a reduction in T-bet expression levels could release GATA3 from sequestration and allow re-activation of T_H_2 genes. Consistent with this possibility, we found that reducing the level of T-bet relative to GATA3 reduces the extent to which GATA3 is redistributed.

We have demonstrated that the redistribution of GATA3 to T-bet binding sites requires T-bet DNA binding function but not GATA3 DNA binding function. This suggests that GATA3 is tethered to T-bet binding sites by T-bet rather than directly contacting the DNA itself. However, although T-box motifs were the only motifs that exhibited stronger enriched at T-bet-dependent GATA3 binding sites compared with invariant GATA3 binding sites, we cannot rule out the possibility that an additional T-bet-dependent factor is also required for GATA3 redistribution. Why T-bet only results in the removal of GATA3 from some of its binding sites (‘loss’ sites) and not others (‘invariant sites’) is unclear, but the reason may be that invariant sites are co-occupied by T-bet whereas loss sites are not.

Our data further demonstrate that the interaction of T-bet with GATA3 requires the same residues that T-bet uses to bind DNA. This is consistent with previous findings that TBX5 and GATA4 mutants which lack the capacity to interact with the other factor also display deficient DNA binding activities (81,83,84,87–89). These data demonstrate that the DNA binding domains of transcription factors do not only govern the DNA binding specificity of the proteins but can also mediate interaction with other transcription factors. This dual function of the T-bet DBD raises the question of how the protein can interact with GATA3 and simultaneously recruit it to DNA. The answer may lie in the ability of T-bet to dimerise (86), with one monomer potentially interacting with DNA and the second monomer interacting with GATA3 (Figure 7).

Transcription factor redistribution through the action of another factor is likely to be a common mechanism in T cell differentiation and beyond. In addition to interacting with GATA3, T-bet directly interacts with the T_FH_ LD-TF BCL6 and targets this factor to a subset of T-bet DNA binding elements in committed T_H_1 cells (38). However, unlike its sequestration of GATA3, T-bet utilises BCL6 to repress expression of genes to which it is recruited. In addition to the altered GATA3 binding profile we have observed in human T_H_1 cells (29), GATA3 exhibits alternative binding profiles at different stages of thymocyte development (90,91), suggesting it can be redistributed by other factors in addition to T-bet. Furthermore, interactions also occur between other T-box and GATA family members, notably TBX5 and GATA4 during heart development (81,92), suggesting that the mechanism we describe for T-bet and GATA3 may be common to co-expressed T-box and GATA factors. Interestingly, in induced pluripotent stem (iPS) cells expressing a GATA4 G296S mutant that disrupts interaction with TBX5, TBX5 is lost from super-enhancers of cardiac genes and instead becomes mislocalized in the genome, resulting in disruption of the cardiac gene program (88). Thus, GATA4 may redistribute TBX5 during cardiomyocyte differentiation. The weak binding of T-bet to GATA3 binding sites that we observed when T-bet was expressed at low levels suggests that there may also be physiological contexts in which GATA3 can alter T-bet's DNA binding profile.

Redistribution of one transcription factor from its canonical binding sites to a different set of sites by another transcription factor may contribute to the widely observed deviation between DNA sequence motifs and transcription factor occupancy and to the differences in the patterns of transcription factor occupancy between cell types or development stages. The redistribution of FLI1 from GATA sites by RUNX1 during embryonic stem cell differentiation (93), the redistribution of SATB1, GATA3 and RUNX1 by PU.1 (94) and RUNX1 by BCL11B (95,96) during thymocyte development provide additional examples of how redistribution of one transcription factor by another can drive gene expression changes that alter cell state.

In summary, we have demonstrated that T-bet directly redistributes GATA3 from its canonical binding sites at T_H_2 genes to T-bet binding sites at T_H_1 genes and that this is associated with suppression of the T_H_2 gene expression program. We propose that this mechanism allows T-bet to supress GATA3 function in cells in which the two LD-TFs are co-expressed and that similar mechanisms may underlie the interplay between other pairs of co-expressed LD-TFs during cell differentiation and development.

## DATA AVAILABILITY

All raw and processed ChIP-seq, RNA-seq and ATAC-seq data reported in this study have been deposited in the National Center for Biotechnology Information (NCBI) Gene Expression Omnibus under accession number GSE171410. Accession numbers of previously published T-bet and GATA3 ChIP-seq data sets are: GSM836124, GSE62486 and GSE109109 (57,63,64). Raw flow cytometry data files have been deposited at the FlowRepository under repository ID FR-FCM-Z3VC.

## Supplementary Material

gkac258_Supplemental_File

## Notes

Present address: Arnulf Hertweck, Nucleome Therapeutics Limited, Oxford, OX3 7FZ, UK.

Present address: Robert Dagil, Centre for Medical Parasitology at Department of Immunology and Microbiology, University of Copenhagen, 2200 Copenhagen N, Denmark.

## References

[1] Fang D. , Zhu J. Dynamic balance between master transcription factors determines the fates and functions of CD4 T cell and innate lymphoid cell subsets. J. Exp. Med. 2017; 214:1861–1876. 28630089 10.1084/jem.20170494 PMC5502437

[2] Huang S. , Guo Y.P., May G., Enver T. Bifurcation dynamics in lineage-commitment in bipotent progenitor cells. Dev. Biol. 2007; 305:695–713. 17412320 10.1016/j.ydbio.2007.02.036

[3] Orkin S.H. Diversification of haematopoietic stem cells to specific lineages. Nat. Rev. Genet. 2000; 1:57–64. 11262875 10.1038/35049577

[4] Evans C.M. , Jenner R.G. Transcription factor interplay in T helper cell differentiation. Brief. Funct. Genomics. 2013; 12:499–511. 23878131 10.1093/bfgp/elt025 PMC3838196

[5] Sungnak W. , Wang C., Kuchroo V.K. Multilayer regulation of CD4 T cell subset differentiation in the era of single cell genomics. Adv. Immunol. 2019; 141:1–31. 30904130 10.1016/bs.ai.2018.12.001

[6] Mirlekar B. Co-expression of master transcription factors determines CD4+ T cell plasticity and functions in auto-inflammatory diseases. Immunol. Lett. 2020; 222:58–66. 32220615 10.1016/j.imlet.2020.03.007

[7] Zhu J. , Yamane H., Paul W.E. Differentiation of effector CD4+ T cell populations. Annu. Rev. Immunol. 2010; 28:445–489. 20192806 10.1146/annurev-immunol-030409-101212 PMC3502616

[8] Weinmann A.S. Roles for helper T cell lineage-specifying transcription factors in cellular specialization. Adv. Immunol. 2014; 124:171–206. 25175776 10.1016/B978-0-12-800147-9.00006-6

[9] Zhu J. T helper cell differentiation, heterogeneity, and plasticity. *Cold S**pring H**arb. P**erspec**t*. Biol. 2018; 10:a030338. 10.1101/cshperspect.a030338 PMC6169815 28847903

[10] Saravia J. , Chapman N.M., Chi H. Helper T cell differentiation. Cell. Mol. Immunol. 2019; 16:634–643. 30867582 10.1038/s41423-019-0220-6 PMC6804569

[11] Spinner C.A. , Lazarevic V. Transcriptional regulation of adaptive and innate lymphoid lineage specification. Immunol. Rev. 2021; 300:65–81. 33615514 10.1111/imr.12935

[12] Butcher M.J. , Zhu J. Recent advances in understanding the Th1/Th2 effector choice. Fac. Rev. 2021; 10:30. 33817699 10.12703/r/10-30 PMC8009194

[13] Hirahara K. , Nakayama T. CD4+ T-cell subsets in inflammatory diseases: beyond the Th1/Th2 paradigm. Int. Immunol. 2016; 28:163–171. 26874355 10.1093/intimm/dxw006 PMC4889886

[14] Zhu X. , Zhu J. CD4 T helper cell subsets and related human immunological disorders. Int. J. Mol. Sci. 2020; 21:8011. 10.3390/ijms21218011 PMC7663252 33126494

[15] Veldhoen M. The role of T helper subsets in autoimmunity and allergy. Curr. Opin. Immunol. 2009; 21:606–611. 19683910 10.1016/j.coi.2009.07.009

[16] Yang R. , Mele F., Worley L., Langlais D., Rosain J., Benhsaien I., Elarabi H., Croft C.A., Doisne J.M., Zhang P. et al. Human T-bet governs innate and Innate-like adaptive IFN-γ immunity against mycobacteria. Cell. 2020; 183:1826–1847. 33296702 10.1016/j.cell.2020.10.046 PMC7770098

[17] Szabo S.J. , Kim S.T., Costa G.L., Zhang X., Fathman C.G., Glimcher L.H. A novel transcription factor, T-bet, directs Th1 lineage commitment. Cell. 2000; 100:655–669. 10761931 10.1016/s0092-8674(00)80702-3

[18] Djuretic I.M. , Levanon D., Negreanu V., Groner Y., Rao A., Ansel K.M. Erratum: transcription factors T-bet and Runx3 cooperate to activate Ifng and silence Il4 in T helper type 1 cells. Nat. Immunol. 2007; 8:145–153. 17195845 10.1038/ni1424

[19] Zhu J. , Jankovic D., Oler A.J., Wei G., Sharma S., Hu G., Guo L., Yagi R., Yamane H., Punkosdy G. et al. The transcription factor T-bet is induced by multiple pathways and prevents an endogenous Th2 cell program during Th1 cell responses. Immunity. 2012; 37:660–673. 23041064 10.1016/j.immuni.2012.09.007 PMC3717271

[20] Zheng W.P. , Flavell R.A. The transcription factor GATA-3 is necessary and sufficient for Th2 cytokine gene expression in CD4 T cells. Cell. 1997; 89:587–596. 9160750 10.1016/s0092-8674(00)80240-8

[21] Pai S.Y. , Truitt M.L., Ho I.C. GATA-3 deficiency abrogates the development and maintenance of t helper type 2 cells. Proc. Natl. Acad. Sci. U.S.A. 2004; 101:1993–1998. 14769923 10.1073/pnas.0308697100 PMC357040

[22] Zhu J. , Min B., Hu-Li J., Watson C.J., Grinberg A., Wang Q., Killeen N., Urban J.F., Guo L., Paul W.E. Conditional deletion of Gata3 shows its essential function in TH1-TH2 responses. Nat. Immunol. 2004; 5:1157–1165. 15475959 10.1038/ni1128

[23] Hwang E.S. , Szabo S.J., Schwartzberg P.L., Glimcher L.H. T helper cell fate specified by kinase-mediated interaction of T-bet with GATA-3. Science. 2005; 307:430–433. 15662016 10.1126/science.1103336

[24] Szabo S.J. , Sullivan B.M., Sternmann C., Satoskar A.R., Sleckman B.P., Glimcher L.H. Distinct effects of T-bet in Th1 lineage commitment and IFN-γ production in CD4 and CD8 T cells. Science. 2002; 295:338–342. 11786644 10.1126/science.1065543

[25] Lee H.J. , Takemoto N., Kurata H., Kamogawa Y., Miyatake S., O’Garra A., Arai N. GATA-3 induces T helper cell type 2 (Th2) cytokine expression and chromatin remodeling in committed Th1 cells. J. Exp. Med. 2000; 192:105–115. 10880531 10.1084/jem.192.1.105 PMC1887713

[26] Zhang D.H. , Cohn L., Ray P., Bottomly K., Ray A. Transcription factor GATA-3 is differentially expressed in murine Th1 and Th2 cells and controls Th2-specific expression of the interleukin-5 gene. J. Biol. Chem. 1997; 272:21597–21603. 9261181 10.1074/jbc.272.34.21597

[27] Ouyang W. , Ranganath S.H., Weindel K., Bhattacharya D., Murphy T.L., Sha W.C., Murphy K.M. Inhibition of Th1 development mediated by GATA-3 through an IL-4- independent mechanism. Immunity. 1998; 9:745–755. 9846495 10.1016/s1074-7613(00)80671-8

[28] Usui T. , Preiss J.C., Kanno Y., Zheng J.Y., Bream J.H., O’Shea J.J., Strober W Erratum: T-bet regulates Th1 responses through essential effects on GATA-3 function rather than on IFNG gene acetylation and transcription. J. Exp. Med. 2006; 203:755–766. 16520391 10.1084/jem.20052165 PMC2118252

[29] Kanhere A. , Hertweck A., Bhatia U., Gökmen M.R., Perucha E., Jackson I., Lord G.M., Jenner R.G. T-bet and GATA3 orchestrate Th1 and Th2 differentiation through lineage-specific targeting of distal regulatory elements. Nat. Commun. 2012; 3:1268. 23232398 10.1038/ncomms2260 PMC3535338

[30] Ouyang W. , Löhning M., Gao Z., Assenmacher M., Ranganath S., Radbruch A., Murphy K.M. Stat6-independent GATA-3 autoactivation directs IL-4-independent Th2 development and commitment. Immunity. 2000; 12:27–37. 10661403 10.1016/s1074-7613(00)80156-9

[31] Cousins D.J. , Lee T.H., Staynov D.Z. Cytokine coexpression during human Th1/Th2 cell differentiation: direct evidence for coordinated expression of Th2 cytokines. J. Immunol. 2002; 169:2498–2506. 12193719 10.4049/jimmunol.169.5.2498

[32] Jenner R.G. , Townsend M.J., Jackson I., Sun K., Bouwman R.D., Young R.A., Glimcher L.H., Lord G.M. The transcription factors T-bet and GATA-3 control alternative pathways of T-cell differentiation through a shared set of target genes. Proc. Natl. Acad. Sci. U.S.A. 2009; 106:17876–17881. 19805038 10.1073/pnas.0909357106 PMC2764903

[33] Messi M. , Giacchetto I., Nagata K., Lanzavecchia A., Natoli G., Sallusto F. Memory and flexibility of cytokine gene expression as separable properties of human TH1 and TH2 lymphocytes. Nat. Immunol. 2003; 4:78–86. 12447360 10.1038/ni872

[34] Hegazy A.N. , Peine M., Helmstetter C., Panse I., Fröhlich A., Bergthaler A., Flatz L., Pinschewer D.D., Radbruch A., Löhning M. Interferons direct Th2 cell reprogramming to generate a stable GATA-3+T-bet+ cell subset with combined Th2 and Th1 cell functions. Immunity. 2010; 32:116–128. 20079668 10.1016/j.immuni.2009.12.004

[35] Peine M. , Rausch S., Helmstetter C., Fröhlich A., Hegazy A.N., Kühl A.A., Grevelding C.G., Höfer T., Hartmann S., Löhning M. Stable T-bet+GATA-3+ Th1/Th2 hybrid cells arise in vivo, can develop directly from naive precursors, and limit immunopathologic inflammation. PLoS Biol. 2013; 11:e1001633. 23976880 10.1371/journal.pbio.1001633 PMC3747991

[36] Bock C.N. , Babu S., Breloer M., Rajamanickam A., Boothra Y., Brunn M.L., Kühl A.A., Merle R., Löhning M., Hartmann S. et al. Th2/1 hybrid cells occurring in murine and human strongyloidiasis share effector functions of Th1 cells. Front. Cell. Infect. Microbiol. 2017; 7:261. 28676845 10.3389/fcimb.2017.00261 PMC5476698

[37] Affinass N. , Zhang H., Löhning M., Hartmann S., Rausch S. Manipulation of the balance between Th2 and Th2/1 hybrid cells affects parasite nematode fitness in mice. Eur. J. Immunol. 2018; 48:1958–1964. 30267404 10.1002/eji.201847639

[38] Oestreich K.J. , Huang A.C., Weinmann A.S. The lineage-defining factors T-bet and Bcl-6 collaborate to regulate Th1 gene expression patterns. J. Exp. Med. 2011; 208:1001–1013. 21518797 10.1084/jem.20102144 PMC3092354

[39] Wang Y. , Godec J., Ben-Aissa K., Cui K., Zhao K., Pucsek A.B., Lee Y.K., Weaver C.T., Yagi R., Lazarevic V. The transcription factors T-bet and Runx are required for the ontogeny of pathogenic interferon-γ-producing T helper 17 cells. Immunity. 2014; 40:355–366. 24530058 10.1016/j.immuni.2014.01.002 PMC3965587

[40] Koch M.A. , Tucker-Heard G., Perdue N.R., Killebrew J.R., Urdahl K.B., Campbell D.J. The transcription factor T-bet controls regulatory T cell homeostasis and function during type 1 inflammation. Nat. Immunol. 2009; 10:595–602. 19412181 10.1038/ni.1731 PMC2712126

[41] Yu F. , Sharma S., Edwards J., Feigenbaum L., Zhu J. Dynamic expression of transcription factors T-bet and GATA-3 by regulatory T cells maintains immunotolerance. Nat. Immunol. 2015; 16:197–206. 25501630 10.1038/ni.3053 PMC4297509

[42] Sefik E. , Geva-Zatorsky N., Oh S., Konnikova L., Zemmour D., McGuire A.M., Burzyn D., Ortiz-Lopez A., Lobera M., Yang J. et al. Individual intestinal symbionts induce a distinct population of RORγ+ regulatory T cells. Science. 2015; 349:993–997. 26272906 10.1126/science.aaa9420 PMC4700932

[43] Ohnmacht C. , Park J.H., Cording S., Wing J.B., Atarashi K., Obata Y., Gaboriau-Routhiau V., Marques R., Dulauroy S., Fedoseeva M. et al. The microbiota regulates type 2 immunity through RORγt+ T cells. Science. 2015; 349:989–993. 26160380 10.1126/science.aac4263

[44] Chung Y. , Tanaka S., Chu F., Nurieva R.I., Martinez G.J., Rawal S., Wang Y.H., Lim H., Reynolds J.M., Zhou X.H. et al. Follicular regulatory T cells expressing Foxp3 and Bcl-6 suppress germinal center reactions. Nat. Med. 2011; 17:983–988. 21785430 10.1038/nm.2426 PMC3151340

[45] Wollenberg I. , Agua-Doce A., Hernández A., Almeida C., Oliveira V.G., Faro J., Graca L. Regulation of the germinal center reaction by Foxp3 + follicular regulatory T cells. J. Immunol. 2011; 187:4553–4560. 21984700 10.4049/jimmunol.1101328

[46] Linterman M.A. , Pierson W., Lee S.K., Kallies A., Kawamoto S., Rayner T.F., Srivastava M., Divekar D.P., Beaton L., Hogan J.J. et al. Foxp3+ follicular regulatory T cells control the germinal center response. Nat. Med. 2011; 17:975–982. 21785433 10.1038/nm.2425 PMC3182542

[47] Tartar D.M. , VanMorlan A.M., Wan X., Guloglu F.B., Jain R., Haymaker C.L., Ellis J.S., Hoeman C.M., Cascio J.A., Dhakal M. et al. FoxP3 + RORγt + T helper intermediates display suppressive function against autoimmune diabetes. J. Immunol. 2010; 184:3377–3385. 20181889 10.4049/jimmunol.0903324 PMC2843758

[48] Lochner M. , Peduto L., Cherrier M., Sawa S., Langa F., Varona R., Riethmacher D., Si-Tahar M., di Santo J.P., Eberl G. In vivo equilibrium of proinflammatory IL-17+ and regulatory IL-10+ Foxp3+ RORγt+ T cells. J. Exp. Med. 2008; 205:1381–1393. 18504307 10.1084/jem.20080034 PMC2413035

[49] Hall A.O.H. , Beiting D.P., Tato C., John B., Oldenhove G., Lombana C.G., Pritchard G.H., Silver J.S., Bouladoux N., Stumhofer J.S. et al. The cytokines interleukin 27 and Interferon-γ promote distinct Treg cell populations required to limit infection-induced pathology. Immunity. 2012; 37:511–523. 22981537 10.1016/j.immuni.2012.06.014 PMC3477519

[50] Oldenhove G. , Bouladoux N., Wohlfert E.A., Hall J.A., Chou D., dos santos L., O’Brien S., Blank R., Lamb E., Natarajan S. et al. Decrease of Foxp3+ Treg cell number and acquisition of effector cell phenotype during lethal infection. Immunity. 2009; 31:772–786. 19896394 10.1016/j.immuni.2009.10.001 PMC2814877

[51] Stock P. , Akbari O., Berry G., Freeman G.J., DeKruyff R.H., Umetsu D.T. Induction of T helper type 1-like regulatory cells that express Foxp3 and protect against airway hyper-reactivity. Nat. Immunol. 2004; 5:1149–1156. 15448689 10.1038/ni1122

[52] Rudra D. , Deroos P., Chaudhry A., Niec R.E., Arvey A., Samstein R.M., Leslie C., Shaffer S.A., Goodlett D.R., Rudensky A.Y. Transcription factor Foxp3 and its protein partners form a complex regulatory network. Nat. Immunol. 2012; 13:1010–1019. 22922362 10.1038/ni.2402 PMC3448012

[53] Wohlfert E.A. , Grainger J.R., Bouladoux N., Konkel J.E., Oldenhove G., Ribeiro C.H., Hall J.A., Yagi R., Naik S., Bhairavabhotla R. et al. GATA3 controls Foxp3+ regulatory T cell fate during inflammation in mice. J. Clin. Invest. 2011; 121:4503–4515. 21965331 10.1172/JCI57456 PMC3204837

[54] Wang Y. , Su M.A., Wan Y.Y. An essential role of the transcription factor GATA-3 for the function of regulatory T cells. Immunity. 2011; 35:337–348. 21924928 10.1016/j.immuni.2011.08.012 PMC3182399

[55] Ichiyama K. , Yoshida H., Wakabayashi Y., Chinen T., Saeki K., Nakaya M., Takaesu G., Hori S., Yoshimura A., Kobayashi T. Foxp3 inhibits RORγt-mediated IL-17A mRNA transcription through direct interaction with RORγt. J. Biol. Chem. 2008; 283:17003–17008. 18434325 10.1074/jbc.M801286200

[56] Zhou L. , Lopes J.E., Chong M.M.W., Ivanov I.I., Min R., Victora G.D., Shen Y., Du J., Rubtsov Y.P., Rudensky A.Y. et al. TGF-B-induced Foxp3 inhibits TH17 cell differentiation by antagonizing RORγt function. Nature. 2008; 453:236–240. 18368049 10.1038/nature06878 PMC2597437

[57] Hertweck A. , Evans C.M., Eskandarpour M., Lau J.C.H., Oleinika K., Jackson I., Kelly A., Ambrose J., Adamson P., Cousins D.J. et al. T-bet activates Th1 genes through mediator and the super elongation complex. Cell Rep. 2016; 15:2756–2770. 27292648 10.1016/j.celrep.2016.05.054 PMC4920892

[58] Li H. , Durbin R. Fast and accurate short read alignment with Burrows-Wheeler transform. Bioinformatics. 2009; 25:1754–1760. 19451168 10.1093/bioinformatics/btp324 PMC2705234

[59] Zhang Y. , Liu T., Meyer C.A., Eeckhoute J., Johnson D.S., Bernstein B.E., Nussbaum C., Myers R.M., Brown M., Li W. et al. Model-based analysis of ChIP-Seq (MACS). Genome Biol. 2008; 9:R137. 18798982 10.1186/gb-2008-9-9-r137 PMC2592715

[60] Ross-Innes C.S. , Stark R., Teschendorff A.E., Holmes K.A., Ali H.R., Dunning M.J., Brown G.D., Gojis O., Ellis I.O., Green A.R. et al. Differential oestrogen receptor binding is associated with clinical outcome in breast cancer. Nature. 2012; 481:389–393. 22217937 10.1038/nature10730 PMC3272464

[61] Zhu L.J. , Gazin C., Lawson N.D., Pagès H., Lin S.M., Lapointe D.S., Green M.R. ChIPpeakAnno: a bioconductor package to annotate ChIP-seq and ChIP-chip data. BMC Bioinf. 2010; 11:237. 10.1186/1471-2105-11-237 PMC3098059 20459804

[62] Gökmen M.R. , Dong R., Kanhere A., Powell N., Perucha E., Jackson I., Howard J.K., Hernandez-Fuentes M., Jenner R.G., Lord G.M. Genome-wide regulatory analysis reveals that T-bet controls Th17 lineage differentiation through direct suppression of IRF4. J. Immunol. 2013; 191:5925–5932. 24249732 10.4049/jimmunol.1202254 PMC3858236

[63] Nakayamada S. , Kanno Y., Takahashi H., Jankovic D., Lu K.T., Johnson T.A., Sun H.wei, Vahedi G., Hakim O., Handon R. et al. Early Th1 cell differentiation is marked by a Tfh cell-like transition. Immunity. 2011; 35:919–931. 22195747 10.1016/j.immuni.2011.11.012 PMC3244883

[64] Fang D. , Cui K., Hu G., Gurram R.K., Zhong C., Oler A.J., Yagi R., Zhao M., Sharma S., Liu P. et al. Bcl11b, a novel GATA3-interacting protein, suppresses Th1 while limiting Th2 cell differentiation. J. Exp. Med. 2018; 215:1449–1462. 29514917 10.1084/jem.20171127 PMC5940260

[65] Shen L. , Shao N., Liu X., Nestler E. Ngs.plot: quick mining and visualization of next-generation sequencing data by integrating genomic databases. BMC Genomics. 2014; 15:284. 24735413 10.1186/1471-2164-15-284 PMC4028082

[66] Bray N.L. , Pimentel H., Melsted P., Pachter L. Near-optimal probabilistic RNA-seq quantification. Nat. Biotechnol. 2016; 34:525–527. 27043002 10.1038/nbt.3519

[67] Soneson C. , Love M.I., Robinson M.D. Differential analyses for RNA-seq: transcript-level estimates improve gene-level inferences. F1000Research. 2016; 4:1521. 10.12688/f1000research.7563.1 PMC4712774 26925227

[68] Love M.I. , Huber W., Anders S. Moderated estimation of fold change and dispersion for RNA-seq data with DESeq2. Genome Biol. 2014; 15:550. 25516281 10.1186/s13059-014-0550-8 PMC4302049

[69] Korotkevich G. , Sukhov V., Budin N., Shpak B., Artyomov M.N., Sergushichev A. Fast gene set enrichment analysis. 2021; bioRxiv doi:01 February 2021, preprint: not peer reviewed 10.1101/060012.

[70] Henderson S. , Pullabhatla V., Hertweck A., de Rinaldis E., Herrero J., Lord G.M., Jenner R.G. The Th1 cell regulatory circuitry is largely conserved between human and mouse. Life Sci. Alliance. 2021; 10:e202101075. 10.26508/lsa.202101075 PMC8960437 34531288

[71] Buenrostro J.D. , Giresi P.G., Zaba L.C., Chang H.Y., Greenleaf W.J. Transposition of native chromatin for fast and sensitive epigenomic profiling of open chromatin, DNA-binding proteins and nucleosome position. Nat. Methods. 2013; 10:1213–1218. 24097267 10.1038/nmeth.2688 PMC3959825

[72] Hochreiter B. , Kunze M., Moser B., Schmid J.A. Advanced FRET normalization allows quantitative analysis of protein interactions including stoichiometries and relative affinities in living cells. Sci. Rep. 2019; 9:8233. 31160659 10.1038/s41598-019-44650-0 PMC6547726

[73] Barber P.R. , Tullis I.D.C., Pierce G.P., Newman R.G., Prentice J., Rowley M.I., Matthews D.R., Ameer-Beg S.M., Vojnovic B. The Gray Institute “open” high-content, fluorescence lifetime microscopes. J. Microsc. 2013; 251:154–167. 23772985 10.1111/jmi.12057 PMC3910159

[74] Barber P.R. , Ameer-Beg S.M., Gilbey J., Carlin L.M., Keppler M., Ng T.C., Vojnovic B. Multiphoton time-domain fluorescence lifetime imaging microscopy: practical application to protein-protein interactions using global analysis. J. R. Soc.Interface. 2009; 6:10.1098/rsif.2008.0451.focus.

[75] Heinz S. , Benner C., Spann N., Bertolino E., Lin Y.C., Laslo P., Cheng J.X., Murre C., Singh H., Glass C.K. Simple combinations of lineage-determining transcription factors prime cis-Regulatory elements required for macrophage and B cell identities. Mol. Cell. 2010; 38:576–589. 20513432 10.1016/j.molcel.2010.05.004 PMC2898526

[76] Grant C.E. , Bailey T.L., Noble W.S. FIMO: scanning for occurrences of a given motif. Bioinformatics. 2011; 27:1017–1018. 21330290 10.1093/bioinformatics/btr064 PMC3065696

[77] Soderquest K. , Hertweck A., Giambartolomei C., Henderson S., Mohamed R., Goldberg R., Perucha E., Franke L., Herrero J., Plagnol V. et al. Genetic variants alter T-bet binding and gene expression in mucosal inflammatory disease. PLoS Genet. 2017; 13:e1006587. 28187197 10.1371/journal.pgen.1006587 PMC5328407

[78] Gorer P.A. Studies in antibody response of mice to tumour inoculation. Br. J. Cancer. 1950; 4:372–379. 14801344 10.1038/bjc.1950.36 PMC2007731

[79] Miller S.A. , Huang A.C., Miazgowicz M.M., Brassil M.M., Weinmann A.S. Coordinated but physically separable interaction with H3K27-demethylase and H3K4-methyltransferase activities are required for T-box protein-mediated activation of developmental gene expression. Genes Dev. 2008; 22:2980–2993. 18981476 10.1101/gad.1689708 PMC2577798

[80] Chen G.Y. , Osada H., Santamaria-Babi L.F., Kannagi R. Interaction of GATA-3/T-bet transcription factors regulates expression of sialil Lewis X homing receptors on Th1/Th2 lymphocytes. Proc. Natl. Acad. Sci. U.S.A. 2006; 103:16894–16899. 17075044 10.1073/pnas.0607926103 PMC1629005

[81] Garg V. , Kathiriya I.S., Barnes R., Schluterman M.K., King I.N., Butler C.A., Rothrock C.R., Eapen R.S., Hirayama-Yamada K., Joo K. et al. GATA4 mutations cause human congenital heart defects and reveal an interaction with TBX5. Nature. 2003; 424:443–447. 12845333 10.1038/nature01827

[82] Shinnakasu R. , Yamashita M., Shinoda K., Endo Y., Hosokawa H., Hasegawa A., Ikemizu S., Nakayama T. Critical YxKxHxxxRP motif in the C-terminal region of GATA3 for its DNA binding and function. J. Immunol. 2006; 177:5801–5810. 17056504 10.4049/jimmunol.177.9.5801

[83] Stirnimann C.U. , Ptchelkine D., Grimm C., Müller C.W. Structural basis of TBX5-DNA recognition: the T-box domain in its DNA-bound and -unbound form. J. Mol. Biol. 2010; 400:71–81. 20450920 10.1016/j.jmb.2010.04.052

[84] Fan C. , Liu M., Wang Q. Functional analysis of TBX5 missense mutations associated with Holt-Oram syndrome. J. Biol. Chem. 2003; 278:8780–8785. 12499378 10.1074/jbc.M208120200 PMC1579789

[85] Khalil A. , Tanos R., El-Hachem N., Kurban M., Bouvagnet P., Bitar F., Nemer G. A HAND to TBX5 explains the link between thalidomide and cardiac diseases. Sci. Rep. 2017; 7:1416. 28469241 10.1038/s41598-017-01641-3 PMC5431093

[86] Liu C.F. , Brandt G.S., Hoang Q.Q., Naumova N., Lazarevic V., Hwang E.S., Dekker J., Glimcher L.H., Ringe D., Petsko G.A. Crystal structure of the DNA binding domain of the transcription factor T-bet suggests simultaneous recognition of distant genome sites. Proc. Natl. Acad. Sci. U.S.A. 2016; 113:E6572–E6581. 27791029 10.1073/pnas.1613914113 PMC5087062

[87] Georges R. , Nemer G., Morin M., Lefebvre C., Nemer M. Distinct expression and function of alternatively spliced Tbx5 isoforms in cell growth and differentiation. Mol. Cell. Biol. 2008; 28:4052–4067. 18391012 10.1128/MCB.02100-07 PMC2423137

[88] Ang Y.S. , Rivas R.N., Ribeiro A.J.S., Srivas R., Rivera J., Stone N.R., Pratt K., Mohamed T.M.A., Fu J.D., Spencer C.I. et al. Disease model of GATA4 mutation reveals transcription factor cooperativity in human cardiogenesis. Cell. 2016; 167:1734–1749. 27984724 10.1016/j.cell.2016.11.033 PMC5180611

[89] Ghosh T.K. , Packham E.A., Bonser A.J., Robinson T.E., Cross S.J., Brook J.D. Characterization of the TBX5 binding site and analysis of mutations that cause Holt-Oram syndrome. Hum. Mol. Genet. 2001; 10:1983–1994. 11555635 10.1093/hmg/10.18.1983

[90] Wei G. , Abraham B.J., Yagi R., Jothi R., Cui K., Sharma S., Narlikar L., Northrup D.L., Tang Q., Paul W.E. et al. Genome-wide analyses of transcription factor GATA3-mediated gene regulation in distinct T cell types. Immunity. 2011; 35:299–311. 21867929 10.1016/j.immuni.2011.08.007 PMC3169184

[91] Zhang J.A. , Mortazavi A., Williams B.A., Wold B.J., Rothenberg E.V. Dynamic transformations of genome-wide epigenetic marking and transcriptional control establish T cell identity. Cell. 2012; 149:467–482. 22500808 10.1016/j.cell.2012.01.056 PMC3336965

[92] Luna-Zurita L. , Stirnimann C.U., Glatt S., Kaynak B.L., Thomas S., Baudin F., Samee M.A.H., He D., Small E.M., Mileikovsky M. et al. Complex interdependence regulates heterotypic transcription factor distribution and coordinates cardiogenesis. Cell. 2016; 164:999–1014. 26875865 10.1016/j.cell.2016.01.004 PMC4769693

[93] Gilmour J. , Assi S.A., Noailles L., Lichtinger M., Obier N., Bonifer C. The co-operation of RUNX1 with LDB1, CDK9 and BRD4 drives transcription factor complex relocation during haematopoietic specification. Sci. Rep. 2018; 8:10410. 29991720 10.1038/s41598-018-28506-7 PMC6039467

[94] Hosokawa H. , Ungerbäck J., Wang X., Matsumoto M., Nakayama K.I., Cohen S.M., Tanaka T., Rothenberg E.V. Transcription factor PU.1 represses and activates gene expression in early T cells by redirecting partner transcription factor binding. Immunity. 2018; 48:1119–1134. 29924977 10.1016/j.immuni.2018.04.024 PMC6063530

[95] Hosokawa H. , Romero-Wolf M., Yui M.A., Ungerbäck J., Quiloan M.L.G., Matsumoto M., Nakayama K.I., Tanaka T., Rothenberg E.V. Bcl11b sets pro-T cell fate by site-specific cofactor recruitment and by repressing Id2 and Zbtb16. Nat. Immunol. 2018; 19:1427–1440. 30374131 10.1038/s41590-018-0238-4 PMC6240390

[96] Shin B. , Hosokawa H., Romero-Wolf M., Zhou W., Masuhara K., Tobin V.R., Levanon D., Groner Y., Rothenberg E.V. Runx1 and Runx3 drive progenitor to T-lineage transcriptome conversion in mouse T cell commitment via dynamic genomic site switching. Proc. Natl. Acad. Sci. U.S.A. 2021; 118:e2019655118. 33479171 10.1073/pnas.2019655118 PMC7848575

