# Supplementary material for: The TH1 cell lineage-determining transcription factor T-bet suppresses TH2 gene expression by redistributing GATA3 away from TH2 genes
Source: Nucleic Acids Res. 2022 Apr 19;50(8):4557–73. doi: 10.1093/nar/gkac258 (PMC9071441; doi:10.1093/nar/gkac258)
Supplement: gkac258_Supplemental_File [file gkac258_supplemental_file.pdf]

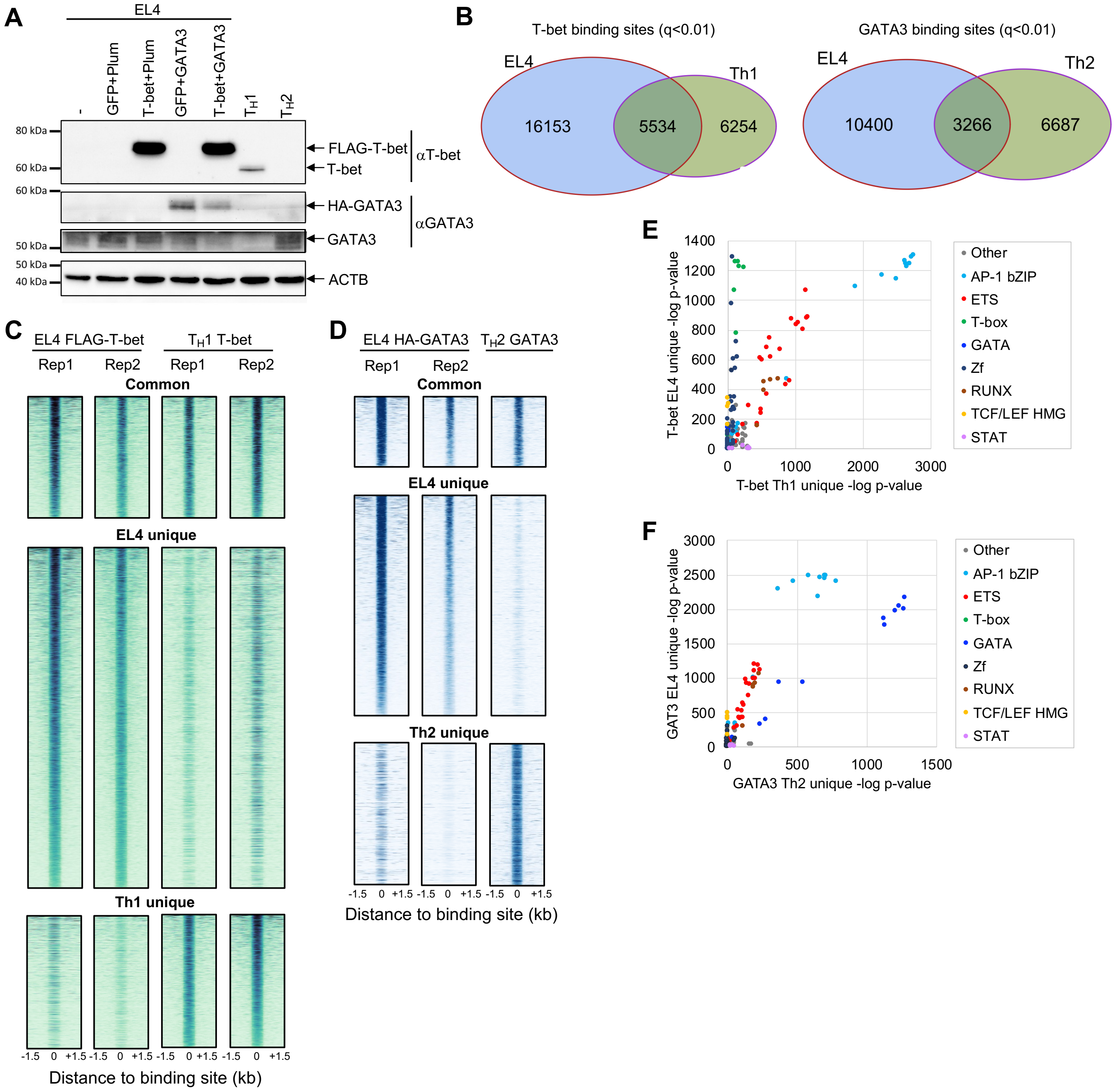

Supplementary Figure 1. Comparison of T-bet and GATA3 occupancy in EL4 cells and T_H cells.
A. Immunoblotting for T-bet and GATA3 in EL4 cells expressing GFP+Plum, GFP+GATA3, T-bet+Plum, T-bet+GATA3 and in primary mouse T_H1 and T_H2 cells. EL4 cells express endogenous *Gata3* but not *Tbx21*.
B. Venn diagrams showing the overlap between T-bet binding sites called by MACS in both EL4 FLAG T-bet and T_H1 cells (top) or in EL4 HA-GATA3 and T_H2 cells (bottom).
C. Heatmaps showing T-bet occupancy at the sets of binding sites shown in A in EL4 cells and T_H1 cells (2 replicates): sites common between EL4 FLAG T-bet and T_H1 cells (top), sites unique to EL4 cells (centre), or sites unique to T_H1 cells (bottom). Sites called by MACS as uniquely bound by T-bet in one cell type or the other often still show evidence of binding in the other cell type suggesting false negatives in the peak calling in one or both replicates.
D. As B, except for GATA3 occupancy in EL4 HA-GATA3 and T_H2 cells.
E. Enrichment of transcription factor binding motifs in the set of sites bound uniquely by T-bet in T_H1 cells or uniquely in EL4 cells. AP-1, ETS and RUNX motifs are strongly enriched at both sets of sites, whereas T-box motifs are more strongly enriched in the set of sites uniquely bound by T-bet in EL4 cells.
F. Enrichment of transcription factor binding motifs in the set of sites bound uniquely by GATA3 in T_H2 cells or uniquely in EL4 cells. GATA, AP-1, ETS and RUNX motifs are strongly enriched at both sets of sites.

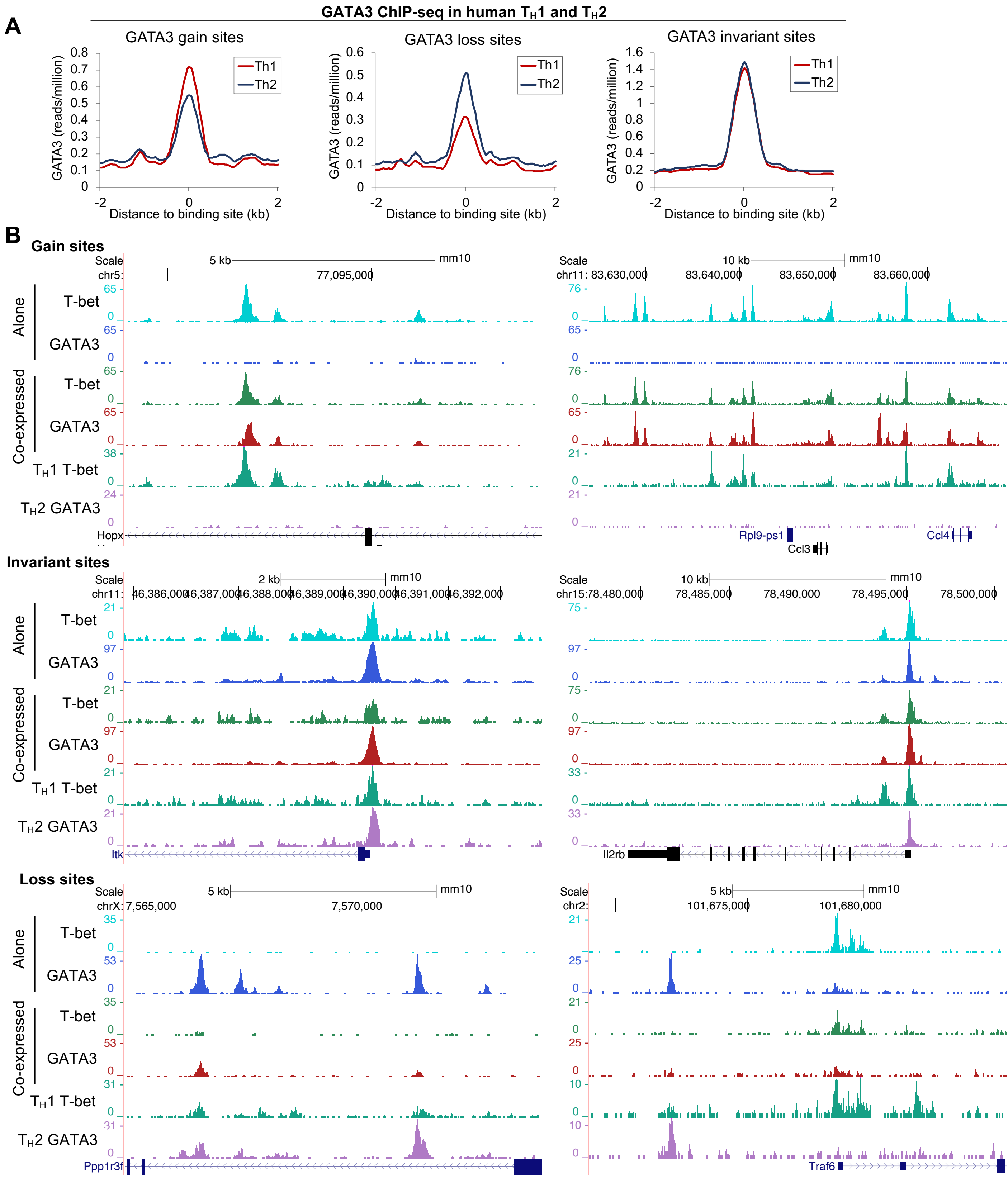

Supplementary Figure 2. Changes in GATA3 occupancy induced by T-bet.

A. Average number of ChIP-seq reads for GATA3 (reads/million) in human T_H1 and T_H2 cells across human genome locations orthologous to GATA3 binding sites that were gained in the presence of T-bet (gain sites), lost in the presence of T-bet (loss sites) or invariant in the presence of T-bet (invariant sites) in mouse EL4 cells.

B. Binding profiles (reads/million) for T-bet and GATA3 at genes associated with GATA3 gain (top), invariant (centre) and loss (bottom) sites in EL4 cells expressing each factor alone or co-expressing the two factors (representative of two biological replicates). T-bet occupancy in mouse T_H1 cells and GATA3 occupancy in mouse T_H2 cells are shown below for comparison.

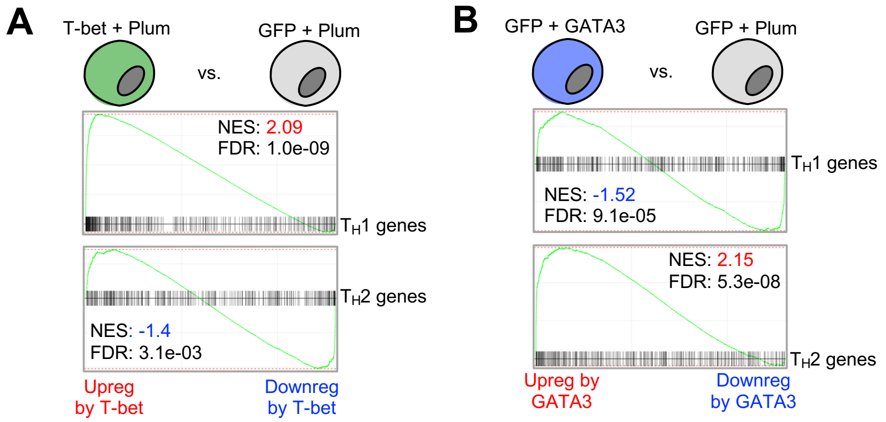

Supplementary Figure 3. T-bet and GATA3 induce their respective gene expression programs in EL4 cells.

A. T-bet induces a T_H1 gene expression program in EL4 cells. Gene set enrichment analysis (GSEA) of T_H1 and T_H2 gene signatures compared with differences in gene expression between cells expressing T-bet and Plum versus GFP and Plum. NES, normalized enrichment score; a positive value indicates enrichment of the gene signature in the set of up-regulated genes and *vice versa*. FDR, false discovery rate.

B. GATA3 induces a T_H2 gene expression program in EL4 cells. Gene set enrichment analysis (GSEA) of T_H1 and T_H2 gene signatures compared with differences in gene expression between cells expressing GFP and GATA3 versus GFP and Plum. Details as for A.

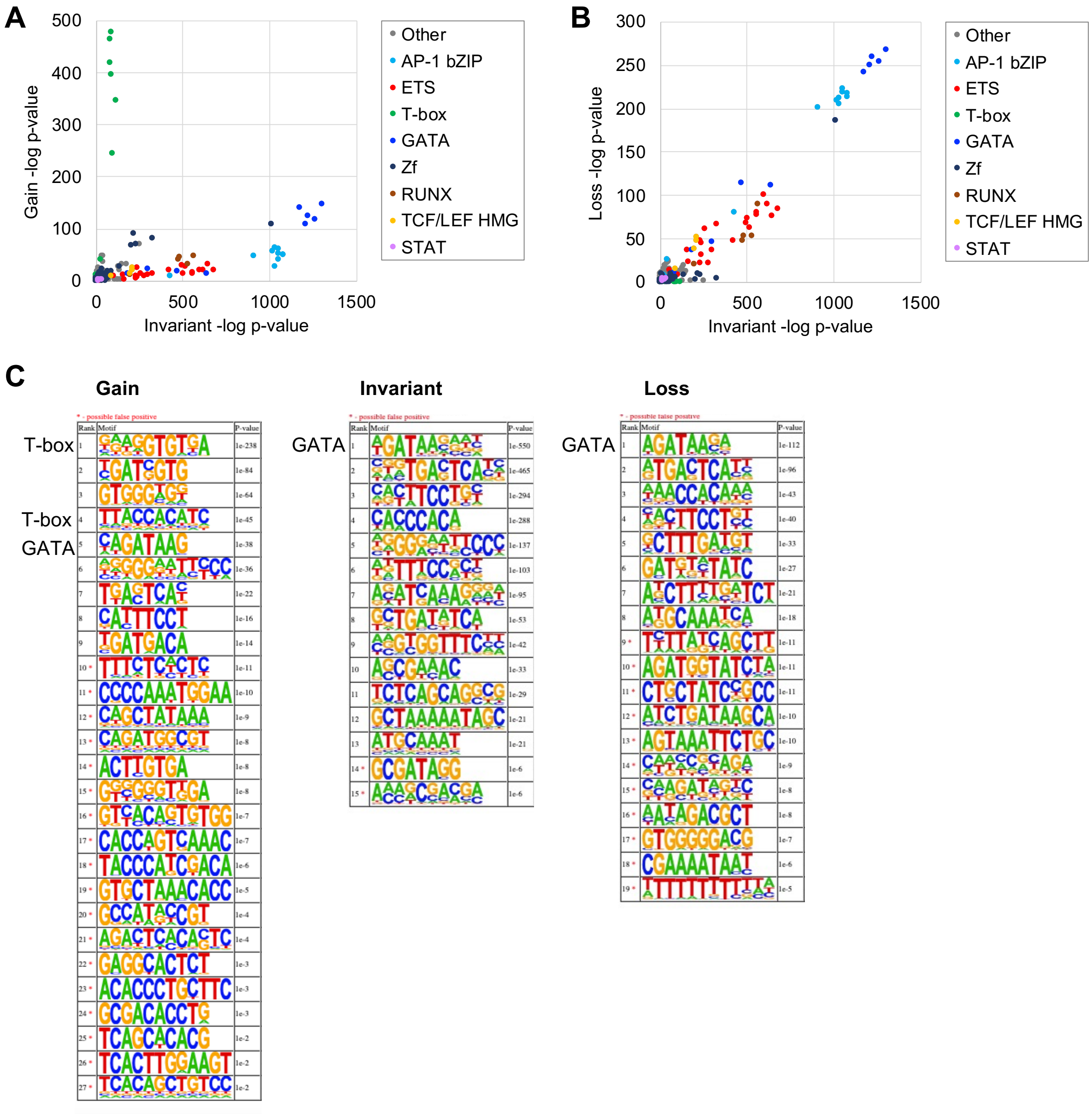

Supplementary Figure 4. Motifs enriched at GATA3 gain, loss and invariant sites.

A. Enrichment (-$\log_{10}$ p-value) of known transcription factor binding motifs at T-bet-dependent GATA3 gain sites compared to T-bet-independent GATA3 invariant sites. T-box motifs are more strongly enriched at GATA3-gain sites.

B. Enrichment (-$\log_{10}$ p-value) of known transcription factor binding motifs at GATA3 binding sites lost in the presence of T-bet (GATA3 loss sites) compared to T-bet-independent GATA3 invariant sites.

C. *De novo*-identified motifs enriched at GATA3 gain, invariant and loss sites, together with their respective p-values. Motifs matching the GATA3 and T-bet consensus motif are highlighted.

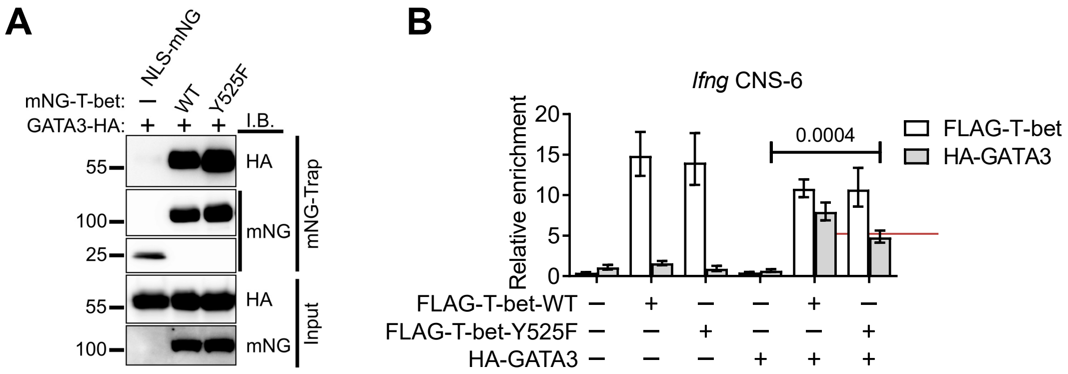

Supplementary Figure 5. T-bet Y525F interacts with GATA3 and recruits GATA3 to gain sites.

A. Immunoblots for mNG and HA-GATA in input samples and mNG immunoprecipitates from HEK293T cells expressing NLS-mNG, mNG-T-bet WT or mNG-T-bet Y525F and GATA3-HA (one biological replicate).

B. Enrichment of *Ifng* CNS-6 DNA relative to input DNA by ChIP for FLAG-T-bet (white) or HA-GATA3 (grey) in EL4 cells expressing WT T-bet or T-bet Y525F in the presence or absence of GATA3 measured by qPCR (mean and SD, n=3 technical replicates of one biological replicate, one-tailed Student's t-test).

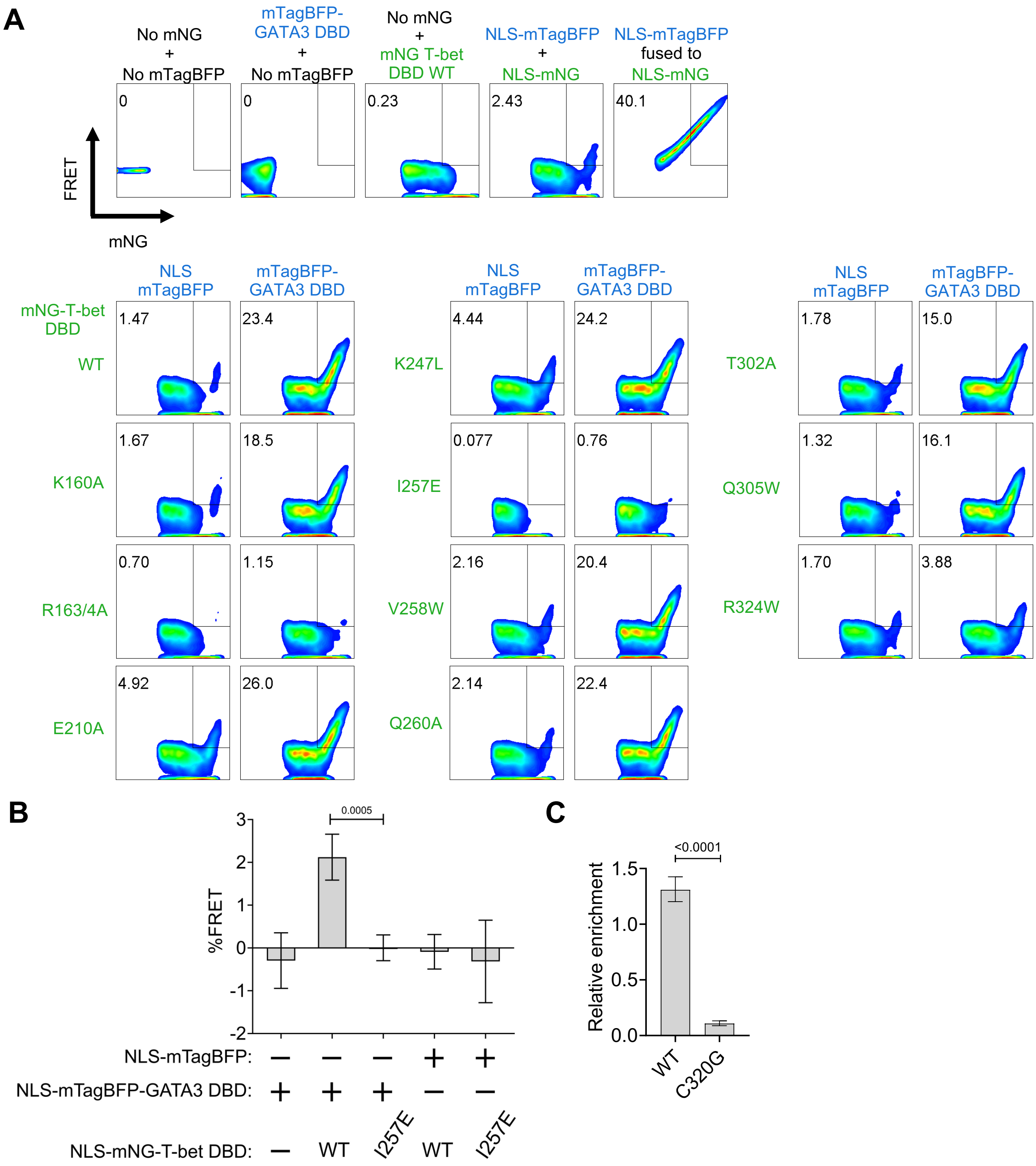

Supplementary Figure 6. Identification of T-bet GATA3 interaction mutants by FRET.

A. Flow cytometric measurement of FRET between mTagBFP-tagged (blue labels) and mNG-tagged (green labels) constructs. The proportion of FRET-positive cells are indicated for each combination of constructs.

B. Percentage of fluorescence energy transferred between mTagBFP-tagged (donor) and mNG-tagged (acceptor) proteins measured by FLIM-FRET (mean and SEM of >= 8 individual cells of one biological replicate, one-tailed Student's t-test).

C. Enrichment of *Il4* CNS DNA by ChIP for WT or C320G HA-GATA3 relative to input DNA in HEK293T cells measured by qPCR (mean and SD, n=3 technical replicates of one biological replicate, one-tailed Student's t-test).
